# A tell tail sign: a conserved C-terminal tail-anchor domain targets a subset of pathogen effectors to the plant endoplasmic reticulum

**DOI:** 10.1093/jxb/erad075

**Published:** 2023-03-01

**Authors:** Emily Breeze, Victoria Vale, Hazel McLellan, Yann Pecrix, Laurence Godiard, Murray Grant, Lorenzo Frigerio

**Affiliations:** School of Life Sciences, University of Warwick, Coventry CV4 7AL, UK; School of Life Sciences, University of Warwick, Coventry CV4 7AL, UK; Division of Plant Science, University of Dundee (at JHI), Invergowrie, Dundee DD2 5DA, UK; CIRAD, UMR PVBMT, Peuplements Végétaux et Bioagresseurs en Milieu Tropical (UMR C53), Ligne Paradis, 97410 St Pierre, La Réunion, France; Laboratoire des Interactions Plantes Microbes Environnement (LIPME), Institut National de Recherche pour l’Agriculture, l’Alimentation, et l’Environnement (INRAE), Centre National de la Recherche Scientifique (CNRS), Université de Toulouse, Castanet-Tolosan, France; School of Life Sciences, University of Warwick, Coventry CV4 7AL, UK; School of Life Sciences, University of Warwick, Coventry CV4 7AL, UK; University of Ghent, Belgium

**Keywords:** Endomembrane, endoplasmic reticulum (ER), oomycete effectors, NAC with Transmembrane Motif1-like (NTL), *Phytophthora infestans*, tail anchor

## Abstract

The endoplasmic reticulum (ER) is the entry point to the secretory pathway and, as such, is critical for adaptive responses to biotic stress, when the demand for *de novo* synthesis of immunity-related proteins and signalling components increases significantly. Successful phytopathogens have evolved an arsenal of small effector proteins which collectively reconfigure multiple host components and signalling pathways to promote virulence; a small, but important, subset of which are targeted to the endomembrane system including the ER. We identified and validated a conserved C-terminal tail-anchor motif in a set of pathogen effectors known to localize to the ER from the oomycetes *Hyaloperonospora arabidopsidis* and *Plasmopara halstedii* (downy mildew of Arabidopsis and sunflower, respectively) and used this protein topology to develop a bioinformatic pipeline to identify putative ER-localized effectors within the effectorome of the related oomycete, *Phytophthora infestans*, the causal agent of potato late blight. Many of the identified *P. infestans* tail-anchor effectors converged on ER-localized NAC transcription factors, indicating that this family is a critical host target for multiple pathogens.

## Teaser

Computational predictions for ER localization identify effectors from eukaryotic phytopathogens with a C-terminal tail-anchor domain, highlighting the importance of the ER as a ubiquitous target for pathogen effector virulence strategies.

## Introduction

The endoplasmic reticulum (ER) is the port of entry of the secretory pathway, acting as the major platform for secretory protein production, transport, folding, and quality control, lipid synthesis, and calcium storage in the cell. Comprised of a highly dynamic, constantly remodelling network of interconnected tubules and flattened cisternae (sheets), the ER extends throughout the cytoplasm and between adjacent cells through plasmodesmata (Hawes *et al.*, 2015; Brandizzi, 2021). The ER also forms heterotypic membrane contact sites with several other organelles including the Golgi, mitochondria, chloroplasts, peroxisomes, and endosomes, together with the plasma membrane, and is continuous with the outer nuclear envelope membrane (Sparkes *et al.*, 2009; Barton *et al.*, 2013; Mehrshahi *et al.*, 2013; Stefano *et al.*, 2015; White *et al.*, 2020). These sites function as specific microdomains for the exchange of molecular cargo and are linked to the propagation of intra- and intercellular signals enabling a coordinated cellular response to internal and external cues (Pérez-Sancho *et al.*, 2016; Wang *et al.*, 2016a; Perico and Sparkes, 2018; Breeze and Mullineaux, 2022).

During pathogen infection, major transcriptional reprogramming occurs, significantly increasing the demand on the protein production and folding machinery (Windram *et al.*, 2012; Lewis *et al.*, 2015). This must be carefully regulated to avoid overloading the ER’s synthetic capacity resulting in the accumulation of proteotoxic unfolded and misfolded proteins, a condition referred to as ER stress (Liu and Howell, 2010, 2016). Cytoprotective signalling programmes, known collectively as the unfolded protein response (UPR), act to mitigate ER stress, but acute or prolonged ER stress will eventually trigger apoptosis programmes, ultimately leading to programmed cell death (PCD) (Srivastava *et al.*, 2018). The ER is a central component facilitating the regulation of adaptive host responses to biotic stress as a consequence of transcriptional reprogramming. Thus, it would not be surprising that successful phytopathogens, regardless of their lifestyle, have evolved ways to target the ER to suppress these immune functions, as well as facilitate the metabolic reconfiguration required to support their nutrition.

Oomycete pathogens such as downy mildews, *Pythium* and *Phytophthora* species, infect a wide range of economically important crop and tree species (Kamoun *et al.*, 2015). During infection, oomycetes form specialized structures called haustoria which act as the delivery site for the secretion of both apoplastic and cytoplasmic effectors, and cell wall-degrading enzymes (Wang *et al.*, 2017). In the early stages of pathogen penetration significant cellular reorganization occurs in the immediate proximity of the haustoria, including the increased association of nuclei and peroxisomes (Boevink *et al.*, 2020), stromule-mediated clustering of chloroplasts (Savage *et al.*, 2021), and accumulation of ER and Golgi (Takemoto *et al.*, 2003; O’Connell and Panstruga, 2006). Indeed, the ER itself may be a major source of the extrahaustorial membrane which separates the pathogen from the host cytosol (Kwaaitaal *et al.*, 2017; Bozkurt and Kamoun, 2020).

Genome-wide studies of multiple oomycete species have revealed that they frequently possess large repertoires (often in excess of 400) of the cytoplasmic Arg-X-Leu-Arg (RXLR) class of effectors (Tyler *et al.*, 2006; Jiang *et al.*, 2008; Haas *et al.*, 2009; Baxter *et al.*, 2010; Sharma *et al.*, 2015). These contain an N-terminal signal peptide targeting the protein for secretion by the pathogen, followed by RXLR and EER motifs that are required for subsequent translocation into the host cell via endocytosis (Whisson *et al.*, 2007; Dou *et al.*, 2008; Kale *et al.*, 2010). However, the precise route by which filamentous eukaryotic cytoplasmic effectors are taken up into the host cell remains unclear and disputed (Petre and Kamoun, 2014). A recent study implicated clathrin-mediated endocytosis in the translocation of fungal (*Magnaporthe oryzae*) effectors into the rice cytoplasm (Oliveira-Garcia *et al.*, 2022, Preprint).

The arsenal of RXLR effectors collectively manipulates multiple host components and signalling pathways to promote virulence (Y. Wang *et al.*, 2019b), with effectors from evolutionarily distinct pathogens frequently converging on the same host target or pathway (Mukhtar *et al.*, 2011; Weßling *et al.*, 2014; McLellan *et al.*, 2022). Whilst the majority of oomycete RXLRs are targeted to the nucleus (or are dually targeted to the nucleus and cytoplasm), a restricted subset also localize to the plasma membrane, endomembrane system, and chloroplasts (Caillaud *et al.*, 2012; Liu *et al.*, 2018; Pecrix *et al.*, 2019; S. Wang *et al.*, 2019; Hossain *et al.*, 2021; Petre *et al.*, 2021).

Very few phytopathogenic effectors have been experimentally validated to localize to the ER, let alone have identified host targets. The *Phytophthora infestans* RXLR effector PITG_03192 has been shown to interact with two potato (*Solanum tuberosum*) NAC transcription factors (TFs) at the ER, preventing their translocation to the host nucleus following treatment with *P. infestans* PAMPs (pathogen-associated molecular patterns) (McLellan *et al.*, 2013). These NACs (StNTP1 and 2) localize to the ER membrane via a transmembrane domain (TMD) which, upon signal perception, is proteolytically cleaved allowing translocation of the cytoplasmic domain to the nucleus (S.-Y. Kim *et al.*, 2006; S.-G. Kim *et al.*, 2010). Arabidopsis contains 14 such annotated NAC with Transmembrane Motif1-like (NTL) TFs. Of these, 12 are validated as being tail anchored to the ER membrane while NTL5/ANAC060 is nuclear localized (Liang *et al.*, 2015) and NTL11/NAC078 shows nucleocytoplasmic localization (Morishita *et al.*, 2009). Besides StNTP1 and 2, other NTLs have also been reported to be targeted by pathogen effectors. These include targeting of NTL9 by the bacterial type III effector HopD1 from *Pseudomonas syringae* (Block *et al.*, 2014) and LsNAC069 from lettuce (an orthologue of StNTP1) by several effectors from the downy mildew *Bremia lactucae* (Meisrimler *et al.*, 2019). These interactions demonstrate target convergence of diverse pathogen effectors from across the *Solanaceae*, *Brassicaceae*, and *Asteraceae* on ER-resident NAC TFs as part of conserved host immune suppression strategies.

Besides preventing the release of ER-located NAC TFs, another pathogen strategy is to deploy effectors that manipulate components of the UPR and thus ER homeostasis. The *Phytophthora sojae* RXLR effector PsAvh262 directly interacts with soybean (*Glycine max*) ER-lumenal Binding immunoglobulin Proteins (BiPs). These ER quality control chaperones are known to be positive regulators of host susceptibility to selected pathogens including *P. sojae* (Jing *et al.*, 2016). PsAvh262 increases pathogen virulence by stabilizing BiPs and ultimately attenuating ER stress-induced PCD. Similarly, PcAvr3a12 from *P. capsici* directly suppresses the activity of an Arabidopsis ER-localized peptidyl-prolyl *cis*-*trans* isomerase (PPIase) involved in protein folding and UPR induction (Fan *et al.*, 2018).

Proteins destined for biological membranes are generally synthesized by cytosolic ribosomes and inserted co-translationally or post-translationally into the appropriate membrane bilayer. In the co-translational pathway, an N-terminal signal peptide on the nascent polypeptide chain is recognized by the signal recognition particle as it emerges from the ribosome and is inserted into the membrane by the Sec61 translocon (Hegde and Keenan, 2011; Voorhees and Hegde, 2016). In contrast, tail-anchored (TA) proteins possess a single TMD close to the C-terminus which serves to target the protein to the correct destination membrane [in combination with the C-terminal sequence (CTS)] and also to anchor the protein in the lipid bilayer (Borgese *et al.*, 2003). Since the hydrophobic TMD in TA proteins only emerges from the ribosomal tunnel after translation is completed and yet must still be shielded from the aqueous environment of the cytosol, this necessitates a post-translational mechanism of membrane insertion. While little is known about the components of the post-translational insertion pathway in plants, the ATP-dependent Guided Entry of Tail (GET) proteins pathway, which catalyses the insertion of TA proteins into the ER, has been well characterized in yeast (Hegde and Keenan, 2011). Furthermore, orthologues of the main GET components have been identified in Arabidopsis and other *Angiospermae* (Srivastava *et al.*, 2017; Xing *et al.*, 2017). TA proteins are also targeted to other organelles, notably the mitochondrial and chloroplast outer membranes and peroxisomes (Rao *et al.*, 2016; Teresinski *et al.*, 2018).

To highlight the ubiquitous nature of ER targeting by effectors from diverse pathogens, we developed and tested bioinformatic predictions of the intracellular distribution of a group of RXLR effectors from three oomycete species: the economically important *P. infestans* (causal agent of potato late blight); *Plasmopara halstedii* (downy mildew of cultivated sunflower, *Helianthus annuus*); and *Hyaloperonospora arabidopsidis* (*Hpa*; downy mildew of *Arabidopsis thaliana*), a model pathosystem for *Peronosporaceae* that infects many major crop species (Kamoun *et al.*, 2015). We found that these RXLR effectors all share a similar protein topology: a C-terminal TMD or tail anchor, which, in the majority of cases, targets them to the ER membrane. We detail a simple and robust *in silico* screening procedure for identifying putative ER-, Golgi-, and mitochondrial-targeted proteins within the effectoromes of sequenced pathogen species and validate a subset of these *in planta*.

## Materials and methods

### Plant materials and growth conditions

*Nicotiana benthamiana* were grown for 5–7 weeks in a compost mix (Levingston F2) in a controlled-environment growth chamber under a 16 h day (21 °C; 120 μmol m^−2^ s^−1^) and 8 h night (18 °C) regime with 60% relative humidity.

### Generation of constructs

*Plasmopara halstedii* effectors were cloned into Gateway vectors as previously described (Pecrix *et al.*, 2019). Candidate TA *P. infestans* effectors (with the exception of PITG_14797 and PITG_10348) were cloned without their signal peptides, as predicted by SignalP (Almagro Armenteros *et al.*, 2019b). Sequences were amplified from *P. infestans* isolate 88069 (Knapova and Gisi, 2002) genomic DNA using gene-specific primers flanked with a portion of the Gateway *att*B recombination sites (all primer sequences are given in Supplementary Table S1). A second round of PCR was performed with full-length *att*B primers, with the resulting *att*B-PCR product purified (Qiagen PCR purification columns) and used to generate an entry clone in pDONRZeo. Entry clones for PITG_14797 and PITG_10348 (minus their predicted signal peptides) were synthesized by Twist Biosciences (San Francisco, USA). N-terminal superfolder green fluorescent protein (sGFP) fusions of the effectors, driven by the constitutive Cauliflower mosaic virus 35S promoter, were created by performing an LR recombination reaction with the Gateway binary destination vector pGWB606 (Nakamura *et al.*, 2014).

### Transient expression

All effector constructs and organellar marker plasmids [red fluorescent protein (RFP)–HDEL (ER), ST–RFP (Golgi), mt-rk (mitochondria) (Nelson *et al.*, 2007)] were transformed via heat shock into *Agrobacterium tumefaciens* strain GV3101 and transiently expressed into *N. benthamiana* leaf epidermal cells using an OD_600_ of 0.2, as previously described (Sparkes *et al.*, 2006). Leaf cells were imaged 3 d after infiltration.

### Microscopy and imaging

#### Confocal microscopy

Freshly excised leaf samples were mounted in water and imaged on a Zeiss LSM 880 confocal microscope with a Plan-Apochromat 63×/1.40 oil DIC M27 objective. GFP was excited at 488 nm and detected in the 498–563 nm range; monomeric RFP (mRFP) was excited at 561 nm and detected in the 602–654 nm range; Chl *a* was excited at 561 nm and detected in the 605–661 nm range.

#### Co-localization analysis

Co-localization scores between effectors and organelle markers were calculated using the co-localization tool in ZEN Blue (Zeiss). A region of interest (ROI) was drawn in each two-channel image and the Costes setting was applied to automatically identify the background threshold (Costes *et al.*, 2004). Pearson’s correlation coefficient for the ROI is reported in the overlay images (Supplementary Figs S1, S2) and is representative of that calculated for *n*=2–7 cells.

### Yeast two-hybrid assays

GAL4 DNA-binding domain fusions were generated for all *P. infestans*, *P. halstedii*, and *H. arabidopsidis* (*Hpa*) effectors in this study by recombination with pDEST32 (Invitrogen) and subsequent transformation of the bait construct into the haploid Y8930 (MATα) yeast strain. A yeast two-hybrid (Y2H) prey library of Arabidopsis NTL/ANAC proteins fused to the GAL4 activation domain (pDEST22; Invitrogen) was similarly created and transformed into the opposite yeast mating strain, Y8800 (MATa). Y2H assays were performed as described in Harvey *et al.* (2020). Empty vectors pDEST22 and pDEST32 transformed into Y8800 and Y8930 yeast strains, respectively, were used as negative controls. Pairwise combinations of effector (bait) and NTL/ANAC (prey) proteins were assessed for growth on selective SD-Leu-Trp-His media, indicative of a protein–protein interaction (PPI). The Y2H assay was repeated three times, and confidence scores were assigned based on a detectable PPI in one (low), two (medium), or three (high) of the biological replicates.

### *In silico* analysis of RXLR effectors

Protein sequences of *P. halstedii*, *Hpa*, and *B. lactucae* effectors were obtained from the NCBI, and that of *P. infestans* from Haas *et al.* (2009). Predictions of the membrane topology of RXLR effectors, notably the relative position and length of the TMD, were performed using both the TMHMM v2.0 (Transmembrane prediction using Hidden Markov Model) (Krogh *et al.*, 2001) and TOPCONS (Tsirigos *et al.*, 2015) algorithms. All annotated *P. infestans* RXLR effector sequences were screened to identify putative TA proteins based on the presence of a single TMD 17–22 residues in length located at the C-terminus with a maximum of 30 residues permitted after the predicted TMD. In fact, over half of the predicted TA effectors identified in this study had <10 residues post-TMD. Evidence for effector expression was obtained from published RNA-Seq data of *P. infestans* infection on tomato (Zuluaga *et al.*, 2016) and potato (Yin *et al.*, 2017), and various life stages of *P. infestans* grown on rye–sucrose agar plates (Ah-Fong *et al.*, 2017).

### Phylogenetic analysis

Protein sequences of Arabidopsis TA NAC/NTL TFs were obtained from the NCBI. Effector and ANAC sequences were aligned using Clustal Omega (Sievers *et al.*, 2011), and phylogenetic trees were generated using iTOL (Interactive Tree of Life) (Letunic and Bork, 2019). Phylogenetic analysis of TA effectors was performed using both the full-length protein sequence (to identify putative orthologues) and the C-terminal region starting 15 amino acids upstream of the predicted tail anchor (to identify putative motifs required for ER, Golgi, or mitochondrial localization).

### Accession numbers

ANAC001, NTL10, AT1G01010; ANAC005, AT1G02250; ANAC013, NTL1, AT1G32870; ANAC014, NTL2, AT1G33060; ANAC016, NTL3, AT1G34180; ANAC017, NTL7, AT1G34190; ANAC040, NTL8, AT2G27300; ANAC053, NTL4, AT3G10500; ANAC060, NTL5, AT3G44290; ANAC062, NTL6, AT3G49530; ANAC068, NTL12, AT4G01540; ANAC069, NTL13, AT4G01550; ANAC078, NTL11, AT5G04410; ANAC086, AT5G17260; ANAC089, NTL14, AT5G22290; ANAC116, NTL9, AT4G35580.

## Results

### C-terminal tail anchor-mediated targeting to the ER membrane is a common strategy employed by oomycete effector proteins

Despite the extensive effector secretome of many oomycetes (Caillaud *et al.*, 2012; Liu *et al.*, 2018; Pecrix *et al.*, 2019; S. Wang *et al.*, 2019; Fabro, 2021), only a relatively small number of effectors have been described that target the host endomembrane system. We therefore sought to broaden this group of ER-localized effectors to facilitate the identification of common host functional pathways targeted by pathogens for immunosuppression. For this we chose the model pathosystem *Hpa* and two economically important oomycete species, *P. infestans* and *P. halstedii* (sunflower downy mildew)—all of which deploy extensive RXLR/RXLR-like effector repertoires.

**Fig. 1.**
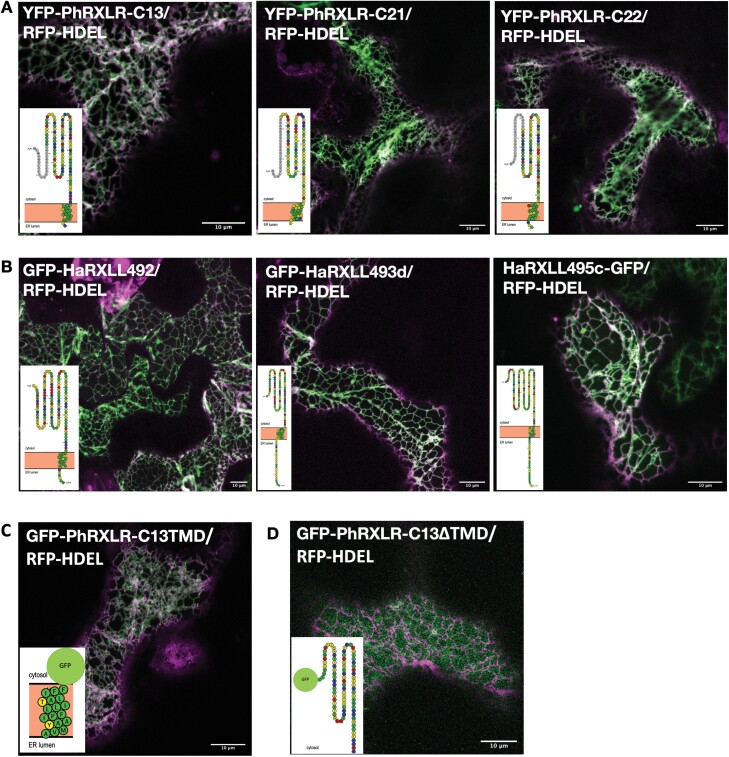
Several ER-localized oomycete effectors possess a C-terminal transmembrane domain (TMD) which is sufficient and necessary for ER localization. Representative merged confocal images of GFP/YFP-tagged effector proteins (green channel) transiently co-expressed with the ER luminal marker RFP–HDEL (magenta channel) in *Nicotiana benthamiana* epidermal cells 3 d after infiltration, with TMHMM-predicted protein topology (inset). (A) *Plasmopara halstedii* (*Ph*) RXLR-C13, C21, and C22. (B) *Hyaloperonospora arabidopsidis* (*Hpa*) RXLL492, 493d, and 495a. (C) PhRXLR-C13TMD_108–125_. (D) PhRXLR-C13∆TMD_108–127_. Scale bars, 10 µm.

In a large-scale screen, Pecrix *et al.* (2019) characterized a number of RXLR effector proteins expressed by *P. halstedii* during infection, of which three, PhRXLR-C13, PhRXLR-C21, and PhRXLR-C22, localized to the ER in *N. benthamiana* and sunflower transient expression assays. We first confirmed these ER localizations (Fig. 1A; Supplementary Fig. S1A; Supplementary Table S2). Despite no significant sequence homology between these three *P. halstedii* effectors, all three are predicted to possess a single TMD positioned towards the C-terminus. Using this observation, we examined the predicted topology of a subset of effectors from the closely related oomycete pathogen *Hpa*, which had been previously characterized as localizing to the ER when expressed *in planta* (Caillaud *et al.*, 2012). Several of these *Hpa* RXLRs also contained putative TMDs at their C-termini (Fig. 1B; Supplementary Fig. S1B; Supplementary Table S2). We thus hypothesized that such TA motifs may represent a common ER-targeting mechanism for oomycete effectors, serving to localize the effector in the ER membrane, with the protein predominating facing the cytosol.

Using the PhRXLR-C13 effector as an exemplar, we tested whether the tail anchor was sufficient for *in planta* effector localization to the ER. GFP was fused directly to a C-terminal fragment of the PhRXLR-C13 effector consisting of the predicted tail anchor (GFP–PhRXLR-C13TMD_108–125_; Fig. 1C; Supplementary Fig. S1C). In addition, a truncated version of the effector lacking the transmembrane-spanning region plus the two C-terminal amino acids at the exoplasmic boundary was also generated (GFP–PhRXLR-C13∆TMD_108–127_; Fig. 1D; Supplementary Fig. S1D). Whilst GFP–PhRXLR-C13TMD_108–125_ showed ER localization comparable with the full-length fusion protein, GFP–PhRXLR-C13 (Fig. 1A), the GFP–PhRXLR-C13∆TMD_108–127_ lacking the TMD was distributed throughout the cytoplasm (Fig. 1D). Hence, the presence of a C-terminal TMD is both necessary and sufficient for the ER localization of the PhRXLR-C13 effector.

### *Phytophthora infestans* has a subset of RXLR effectors with a predicted tail-anchor topology

We hypothesized that other oomycete pathogens may also possess a repertoire of ER-targeted effector proteins sharing a similar tail anchor topology, which could further be employed in an *in silico* prediction tool to identify ER membrane-localized effectors. We performed a stringent bioinformatic analysis of the 563 known RXLR effectors from the oomycete *P. infestans* strain T30-4 (Haas *et al.*, 2009). Alongside *P. halstedii* and *Hpa,* all *Phytophthora* spp. are members of the Peronosporales order, with phylogenetic analyses placing *P. infestans* (together with *P. nicotianae* and *P. parasitica*) in a sister clade to *P. halstedii* (McCarthy and Fitzpatrick, 2017).

We used the membrane topology prediction algorithm TMHMM v2.0 (Krogh *et al.*, 2001) to identify and position any TMDs within the known RXLR effector sequences. TA proteins are inserted post-translationally into their target membrane once the hydrophobic TMD emerges from the ribosome exit tunnel (Hegde and Keenan, 2011). Since this channel is estimated to hold a polypeptide chain of ~30 amino acids, the maximal permitted luminal sequence downstream of the predicted TMD was set to 30 residues (Kriechbaumer *et al.*, 2009). Plant ER-localized transmembrane helices are typically between 17 and 22 residues in length (Brandizzi *et al.*, 2002; Parsons *et al.*, 2019) and thus an effector was defined as being ‘tail-anchored’ if it possessed a predicted TMD within 50 residues of its C-terminus. These stringent criteria identified 17 putative TA *P. infestans* RXLR effectors, hereafter referred to as Group I effectors (Table 1; Supplementary Table S3), and an additional eight potential candidates (Group II effectors) that fell marginally outside these parameters. The latter comprised five effectors with predicted TMDs slightly below the posterior probability cut-off employed by TMHMM and three effectors with C-terminal TMDs but beyond the specified final 50 residues. To test our pipeline parameters, we analysed the protein sequences of five effectors from the oomycete *B. lactucae* (BLN03, BLN04, BLR05, BLR08, and BLR09) which have previously been characterized as tail-anchored and found to localize to the ER and/or interact with the ER-localized LsNAC069 TF (Meisrimler *et al.*, 2019). We were able to identify the TMD in all five *B. lactucae* effectors (Supplementary Table S4).

**Table 1.** Putative tail-anchored *P. infestans* (T30-4 isolate) RXLR effectors

*P. infestans* RXLR ID	Total protein length (amino acids) (T30-4)	Position of predicted TMD (T30-4)	Length of predicted TMD (amino acids) (T30-4)	Evidence of expression^*a*^	Subcellular localization (*N. benthamiana*)	Grand average of TMD hydropathy (GRAVY)
**Group I**						
** PITG_03192**	144	122–139	17	Yes	**ER**	2.43
PITG_04280	200	172–194	22	No	N/A	1.54
** PITG_04367**	184	159–181	22	No	**ER**	1.78
** PITG_09218**	165	126–148	22	Yes	**Mitochondria**	1.16
** PITG_09223**	144	115–137	22	Yes	**ER**	1.81
PITG_09224	140	119–138	19	Yes	N/A	1.92
PITG_10835	242	207–229	22	Yes	N/A	1.51
** PITG_13044**	252	229–251	22	Yes	**ER**	2.05
** PITG_13045**	136	113–135	22	Yes	**ER and Golgi**	2.14
** PITG_13048**	252	229–251	22	Yes	**ER**	1.95
** PITG_14797**	**123**	**97–119**	**22**	**No**	**Golgi**	**1.43**
** PITG_15235**	183	148–170	22	Yes	**Golgi (ER)**	1.65
PITG_15315	134	97–115	18	No	N/A	2.09
PITG_20940	184	159–181	22	No	N/A	1.78
PITG_22868	152	128–150	22	No	N/A	1.54
** PITG_22884**	154	129–151	22	No	**Mitochondria**	1.49
** PITG_23046**	111	78–97	19	Yes	**Golgi**	2.00
**Group II**						
** PITG_15732**	327 (256)	238–261 (238–255)^*b.c*^	21	No	ND (very weak expression)	1.84 (2.33)
PITG_19529	236	172–194^*b*^	22	No	N/A	1.54
** PITG_23202**	136	78–97^*b*^	19	Yes	**Golgi (ER)**	2.18
** PITG_09216**	175	(139–159)^*d*^	20	Yes	**Mitochondria**	1.02
** PITG_10348**	**207**	**(168–188)** ^*d*^	**20**	**No**	**Cytoplasm**	**1.26**
** PITG_15297**	119	(93–111)^*d*^	18	Yes	**ER**	2.38
** PITG_15318**	119	(93–111)^*d*^	18	Yes	**ER**	2.38
PITG_23117	124	(88–106)^*d*^	18	No	N/A	1.98

^*a*^ Zuluaga *et al.* (2016); Ah-Fong *et al.* (2017); Yin *et al.* (2017).

^*b*^ Putative TMD is >50 residues from the C-terminus..

^*c*^ In *P.infestans* 88069 strain, PITG_15732 is truncated relative to *P.infestans* T30-4 strain such that the position of the TMD is located within 50 residues of the C-terminus.

^*d*^ Putative TMD falls below the TMHMM posterior probability cut-off.

**Fig. 2.**
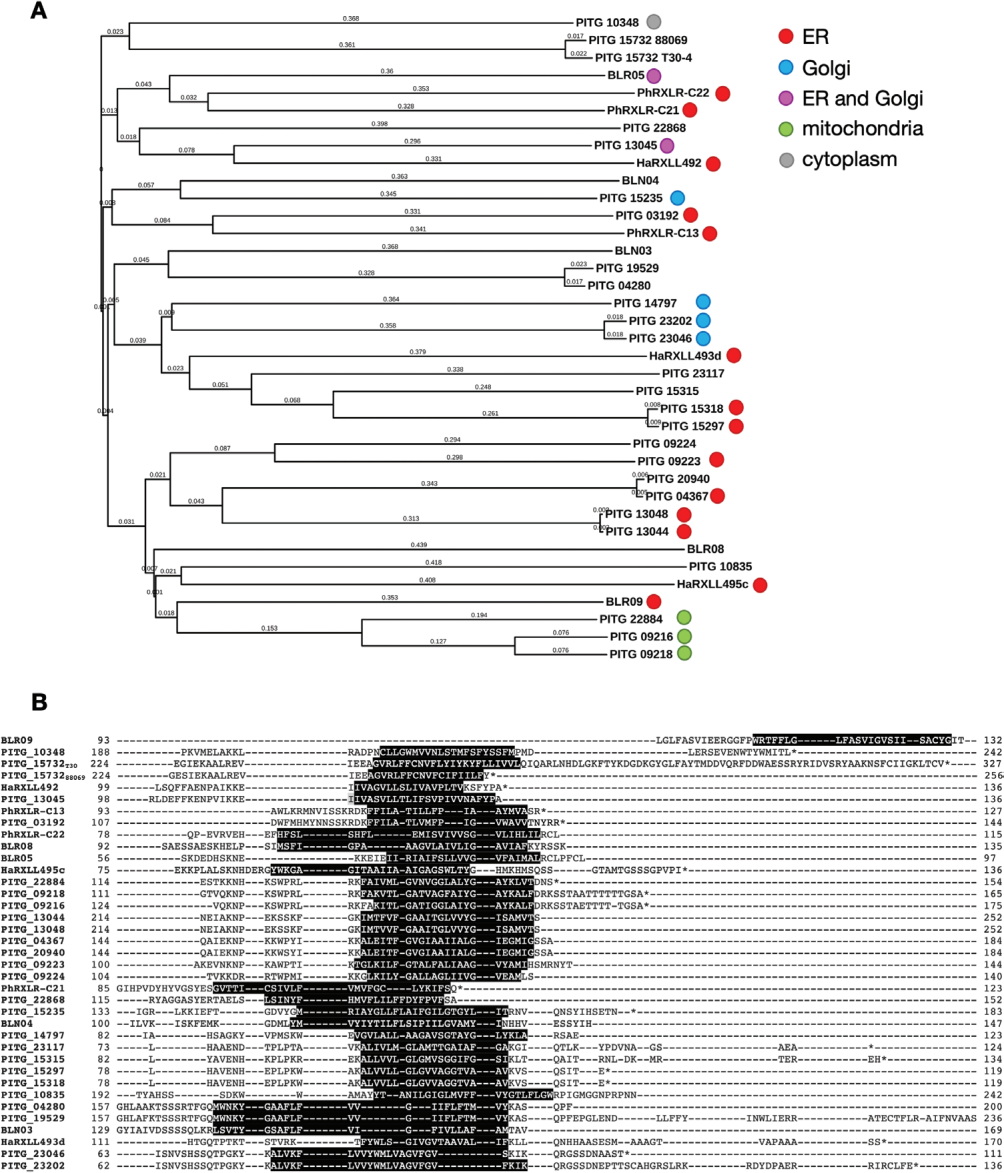
The C-terminal transmembrane domains (TMDs) of tail-anchored effectors are partially conserved between and within oomycete species. (A) Phylogeny of TA effectors from *P. infestans*, *Hpa*, *P. halstedii*, and *B. lactucae* based on whole protein sequences. Filled circles indicate experimentally determined and/or published subcellular localization (McLellan *et al.*, 2013; Meisrimler *et al.*, 2019; Wang *et al.*, 2019a) (red, ER; blue, Golgi; pink, ER and Golgi; green, mitochondria; grey, cytoplasm). (B) Alignment of the C-terminal region of *P. infestans*, *Hpa*, *P. halstedii*, and *B. lactucae* effectors described in this study. Predicted TMDs are highlighted in black.

Phylogenetic analysis of (i) the total protein and (ii) C-terminal regions of these 25 *P. infestans* TA effectors and the previously characterized *Hpa*, *B. lactucae*, and *P. halstedii* ER effectors showed some evidence of intra- and interspecies homology, notably in the C-terminal transmembrane region (Fig. 2). PhRXLR-C13 and the previously characterized PITG_03192 effector (McLellan *et al.*, 2013), for example, have 33% sequence identity across the entire protein sequence and 56% within the TMD. Similarly, HaRxLL492 and PITG_13045 share 37% overall similarity (65% within the TMD).

We selected a subset of *P. infestans* effectors from both Group I and II for further detailed investigation, ensuring coverage of all the identified phylogenetic clades (Fig. 2A). Since the majority of *P. infestans* effectors inventoried are not experimentally validated, we added an additional criterion for evidence of expression during pathogen infection derived from published RNA-Seq data (Zuluaga *et al.*, 2016; Ah-Fong *et al.*, 2017; Yin *et al.*, 2017). Based upon these conditions, we initially cloned 10 high confidence (Group I) TA effectors plus five Group II effectors (minus the N-terminal signal peptide) (Table 1) from the widely used laboratory isolate 88069 of *P. infestans* (Knapova and Gisi, 2002). As a consequence, some of the cloned sequences exhibited minor amino acid substitutions compared with the published T30-4 sequences (Supplementary Table S3), or in the case of PITG_15732 a truncation, resulting in the TMD now being positioned within our previously defined TA region. PITG_15732 is a homologue of the well-characterized *P. sojae* effector Avr3b, both possessing a nudix hydrolase domain which has been shown to contribute to Avr3b-mediated virulence (Dong *et al.*, 2011). While other effectors containing the nudix hydrolase motif are nucleo-cytoplasmic (PITG_06308 and PITG_15679) (S. Wang *et al.*, 2019), the presence of the TMD at the C-terminus of PITG_15732 suggested a possible ER address.

### Tail-anchored effectors localize predominantly to the ER and Golgi

To test if the predicted TA effectors localized to the ER *in planta*, we created constitutively expressed N-terminal fluorescent protein-tagged fusions (minus the pathogen signal peptide to recapitulate delivery into the plant cytosol), such that the predicted topology of the chimeric protein had the GFP moiety orientated to the cytosol. Following transient expression in *N. benthamiana* epidermal cells, subsequent confocal microscopy 3 d after infiltration allowed subcellular visualization of the tagged effectors, the majority of which exhibited strong fluorescent protein expression. We could not detect any expression of the PsAvr3b homologue, PITG_15732.

**Fig. 3.**
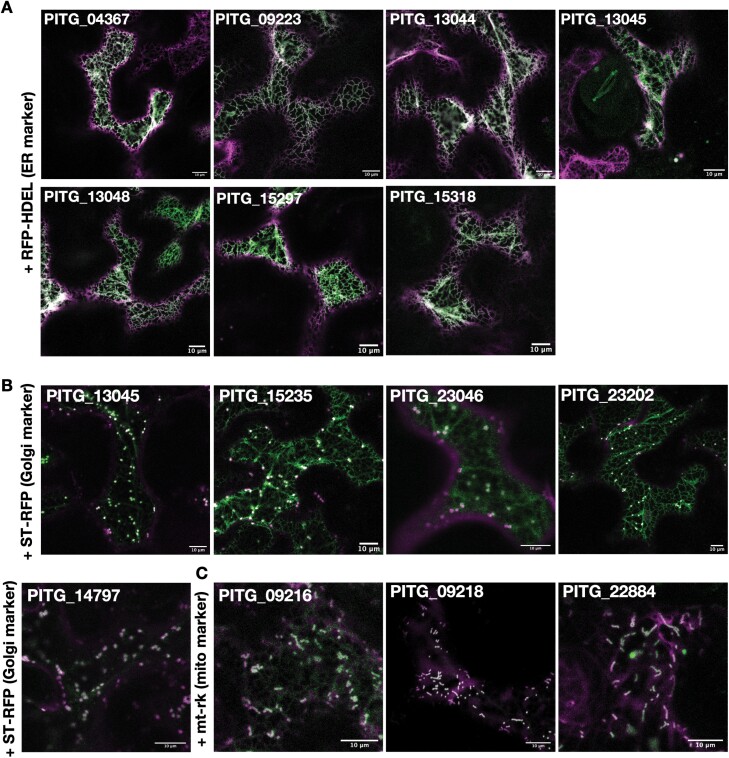
*Phytophthora infestans* tail-anchored RXLR effectors localize to the ER, Golgi, and mitochondria. Representative merged confocal images of 35S:GFP–PITG constructs (green channel) transiently co-expressed in *N. benthamiana* epidermal cells 3 d after infiltration with (A) ER (RFP–HDEL), (B) Golgi (ST–RFP), or (C) mitochondrial markers (mt-rk) (Nelson *et al.*, 2007) (magenta channel). Scale bars, 10µm.

In addition to the six ER-localized tagged *Hpa* and *P. halstedii* effectors (Fig. 1), eight of the 15 putative TA *P. infestans* effectors co-localized with the ER luminal marker RFP–HDEL. PITG_13045 also co-localized with the Golgi marker ST–RFP (Fig. 3A, B; Supplementary Fig. S2A, B). Of those effectors that did not localize to the ER, three effectors, PITG_23202 and its close homologue PITG_23046, and PITG_15235 were predominantly Golgi localized (with faint ER signal). Further, three of the four remaining GFP-tagged *P. infestans* effectors (PITG_09216, PITG_09218, and PITG_22884) co-localized with a mitochondrial marker (Nelson *et al.*, 2007) (Fig. 3C; Supplementary Fig. S2C), as previously described by S. Wang *et al.* (2019), for PITG_09218.

The precise targeting of TA proteins to their destination membrane has been suggested to depend on multiple physiochemical properties of both the TMD and C-terminal regions. These include the length of the TMD and its hydrophobicity, and the overall charge of the CTS distal to the TMD and specific motifs therein (Marty *et al.*, 2014; Rao *et al.*, 2016). Here, the length of the predicted TMD and CTS and the abundance of basic residues in the latter was comparable in the three mitochondrial effectors with those of the ER- and Golgi-localized effectors (Table 1; Supplementary Table S3). Furthermore, although the outer mitochondrial membrane dibasic targeting motif (-R-R/K/H-X^[X≠E]^) (Marty *et al.*, 2014) was present in two of the three mitochondria-localized effectors, it was also present in the ER-localized effector, PITG_23202. Analysis of the effector protein sequences using the TargetP-2.0 server (Almagro Armenteros *et al.*, 2019a), which predicts the presence of mitochondrial (and chloroplast) targeting peptides, did not identify any targeting feature, suggesting that the C-terminal tail anchor is indeed the primary sorting mechanism for these mitochondrial effectors.

The Grand Average of Hydrophobicity (GRAVY) (Kyte and Doolittle, 1982) scores of the *P. infestans* effector TMDs (Table 1) revealed that despite considerable variation in TMD hydrophobicity, the mitochondria-localized proteins had significantly lower values than those of the effectors targeted to the ER [*P*=0.0079; mean GRAVY score: 2.06 (ER-localized effectors), 1.22 (mitochondria-localized effectors)], as previously described (Kriechbaumer *et al.*, 2009; Rao *et al.*, 2016). On the basis of this observation, we hypothesized that PITG_10348 and PITG_14797 may localize to organelles other than the ER since their predicted TMDs had relatively low GRAVY scores of 1.26 and 1.43, respectively. Indeed, PITG_14797 localized to the Golgi (Fig. 3B), as observed for PITG_23202 and PITG_23046 which appear in the same phylogenetic clade as PITG_14797 (Fig. 2A); but PITG_10348 showed a cytoplasmic localization (Supplementary Fig. S2C), corroborating the TMHMM posterior probability cut-off for TMD prediction and inferring that PITG_10348 is not a TA effector.

### Tail-anchored oomycete effectors converge on membrane-tethered NAC TF targets

**Fig. 4.**
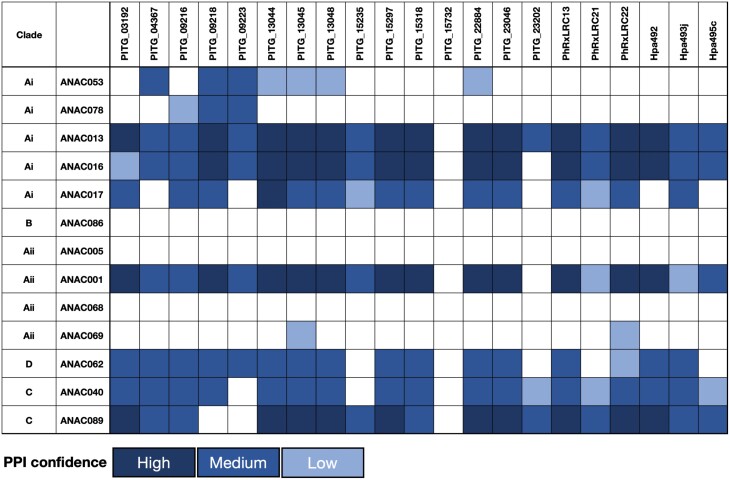
A subset of ER-localized NAC transcription factors (TFs) interact with several tail-anchored oomycete effectors. Protein–protein interactions (PPIs) between NAC TFs and selected *P. infestans*, *Hpa*, and *P. halstedii* effectors were determined by yeast two-hybrid (Y2H) assays. Positive interactions between bait constructs (effector–GAL4-binding domain fusion) and prey constructs (NAC–GAL4 activation domain fusion) resulting in activation of the HIS3 reporter gene were detected by growth on media lacking histidine (SD-Leu-Trp-His). The Y2H assay was performed three times and confidence scores were assigned based on a detected PPI in one (low), two (medium), or three (high) of the biological replicates. Results from one biological replicate and phylogeny of ANACs (NTLs) are shown in Supplementary Fig. S3.

Although the specific host protein(s) targeted by identified ER-localized effectors have been described in only a handful of cases, several effectors from multiple oomycete and bacterial species converge on the plant NTL family of TFs (McLellan *et al.*, 2013; Block *et al.*, 2014; Meisrimler *et al.*, 2019). To determine if our subset of TA effectors were also capable of interacting with membrane-localized NACs, we performed binary yeast Y2H assays with 11 of the 14 identified Arabidopsis NTLs (NTL2/ANAC014, NTL5/ANAC060, and NTL9/ANAC116 were not present in our library) (Fig. 4). ANAC005 and ANAC086 were also included in the Y2H screen since they group phylogenetically with the NTLs despite not containing a predicted TMD (Supplementary Fig. S3A).

Several, but not all, of the *P. infestans*, *Hpa*, and *P. halstedii* effectors showed PPIs with ANAC013 (NTL1), ANAC016 (NTL3), ANAC017 (NTL7), ANAC001 (NTL10) [but not with other members of this phylogenetic clade (Supplementary Fig. S3A)], ANAC062 (NTL6), ANAC040 (NTL8), and ANAC089 (NTL14). No effector interacted with the non-TA ANACs, ANAC005, and ANAC086.

## Discussion

In this study, we first identified a conserved C-terminal TA motif in a subset of previously validated ER-localized effectors from the oomycete pathogens *H. arabidopsidis* and *P. halstedii.* On the basis of the presence of this structural feature, we next developed and validated an *in silico* screen to identify several previously uncharacterized effectors within the effectorome of the oomycete pathogen, *P. infestans*, which are directly targeted to the ER (and Golgi) *in planta*. Finally, we showed that a number of these ER-localized effectors target the NTL TF family. Future studies will investigate other host targets in addition to expanding potential NTL interactions.

Many integral ER membrane proteins possess either a di-arginine or a di-lysine ER retention and retrieval motif (Schutze *et al.*, 1994), whilst soluble ER luminal proteins frequently encode a K/HDEL motif at their C-terminus (Gomord *et al.*, 1997). Using truncated versions of the PhRXLR-C13 effector, we demonstrated here that the TMD alone was necessary and sufficient to localize the protein to the ER membrane.

Tail anchors are also a known sorting mechanism for proteins resident on the outer envelope of plastids, mitochondria, and peroxisomes. Indeed, three of our 16 TA *P. infestans* effectors tested were observed to localize to the mitochondria, including PITG_09218 as previously reported (S. Wang *et al.*, 2019). The hydrophobicity of the TMD largely differentiates between ER and mitochondrial TA effector TMDs, the latter being weakly hydrophobic (GRAVY score <1.5) as previously described in both plant and animal systems (Kriechbaumer *et al.*, 2009; Marty *et al.*, 2014; Rao *et al.*, 2016; Chio *et al.*, 2017). Hydrophobicity parameters could thus be incorporated into future iterations of the *in silico* effector screening pipeline to aid discrimination between likely ER effectors and those localizing to other organelles, but with the caveat that such scores are reliant on the correct prediction of TMD sequence and length. Additional, as yet unidentified criteria, beyond TMD hydrophobicity, would appear to determine localization to the Golgi since PITG_14797, which has a relatively low TMD hydrophobicity (GRAVY score 1.43), is not localized to the mitochondria as we predicted, but is instead trafficked to the Golgi, as are its most closely sequence-related homologues, PITG_23202 and PITG_23046.

Alignment of both the full-length protein sequences and the TMD sequences of the effectors (Fig. 2) did not identify any conserved localization motifs, known or novel, pertaining to ER, Golgi, or mitochondrial localization. We saw no evidence to indicate that basic residues in the CTS help reject TA proteins from the ER or target them instead to the mitochondria (Table 1), as previously demonstrated by Rao *et al.* (2016) in yeast. It would be interesting to extend this sequence analysis to oomycete effectors confirmed as localizing to the chloroplast and/or peroxisomes to further refine these biochemical parameters and clarify the extent of the role of effector-encoded TA proteins in subcellular targeting.

The ER membrane-localized effectors we identified represented only a small proportion of the large number of predicted *P. infestans* RXLR effectors (>500). The majority of tested oomycete effectors localize to the nucleus and/or cytosol, with a smaller number being targeted to the plasma membrane, chloroplasts, and endomembrane system (Caillaud *et al.*, 2012; Khan *et al.*, 2017; Liu *et al.*, 2018; Pecrix *et al.*, 2019; S. Wang *et al.*, 2019; Hossain *et al.*, 2021; Petre *et al.*, 2021). However, alternative mechanisms other than the presence of a tail ancho are highly likely to be employed by effectors targeted to the ER membrane or lumen, including the aforementioned di-Arg/Lys or H/KDEL motifs. Such candidates include PITG_09585 which encodes the terminal KDEL ER retention signal, and the BiP-targeting PsAvh262 (Jing *et al.*, 2016) which does not possess a tail anchor or known ER sequence motifs. It is important to note that early genome-wide effector discovery pipelines frequently excluded proteins with a predicted TMD (Sperschneider *et al.*, 2015). Consequently, it is plausible that there are additional unannotated ER-localized effectors within the genomes of several well-studied pathogen species.

Pathogenic effectors are under strong selective pressure as part of the perpetual evolutionary arms race to avoid detection by host resistance proteins. However, within the *Phytophthora* genus, there is evidence of protein sequence conservation for several effectors, but this is less evident in more distantly related oomycete species. Effector homology is likely to be indicative of conserved functionality, with successful manipulation of the corresponding host target(s) being crucial for pathogenicity (McLellan *et al.*, 2022). Here we identified two pairs of ER-localized effectors from different oomycetes, PhRXLR-C13 and PITG_03192, and HpaRXLL492 and PITG_13045, with some shared sequence homology both outside of, and notably within, their predicted TMDs. Indeed, further orthologues of PhRXLR-C13 and PITG_03192 have also been identified in several other oomycete species including *P. parasitica*, *P. sojae*, and *P. viticola* (Liu *et al.*, 2021). PITG_03192 localizes to the ER in *N. benthamiana* and prevents the relocalization of two host NAC TFs (NTP1 and 2) from the ER to the nucleus with a corresponding impact on *P. infestans* susceptibility (McLellan *et al.*, 2013). Similarly, Meisrimler *et al.* (2019) described the interaction of PITG_03192 with a NAC TF from lettuce (*Lactuca sativa*), LsNAC069. LsNAC069 forms a phylogenetic cluster with StNTP2 and with ANAC013, ANAC016, and ANAC017, which were also found to interact with PITG_03192. Here, we detected PPIs with ANAC013, ANAC016, and ANAC017 for both PITG_03192 and the closely related PhRXLR-C13 effector. Taken together, this supports the hypothesis that a subset of effectors from different pathogens are conserved in both their protein structure and infection strategy (McLellan *et al.*, 2022).

Several of the remaining ER-localized *P. infestans*, *P. halstedii*, and *Hpa* effectors also interacted with ANAC013, ANAC016, and ANAC017 (clade Ai, Supplementary Fig. S3A); ANAC001 (but not with other members of this phylogenetic clade Aii); and with ANAC040 and ANAC089 (clade C), in our Y2H assays. This convergence of multiple effectors from phylogenetically diverse pathogens on a subset of NAC targets, even in non-adapted pathogens, suggests that these TFs are key players in the host defence response. During ER stress, ANAC089, for example, relocates from the ER to the nucleus, promoting the transcriptional up-regulation of genes associated with the UPR and PCD (Yang *et al.*, 2014). Thus, it is possible that the ER quality control system may act as an early sensor and signal transducer of environmental stress conditions, enabling the ER secretory machinery to be primed to meet the increased demand for stress-related proteins (Pastor-Cantizano *et al.*, 2020). Hence, the UPR is critical for adaptive responses to environmental stress, with the direct or indirect manipulation of various components of the UPR pathway by effectors probably representing a common virulence strategy employed by pathogens.

ANAC089 has also been shown to interact with VAP27-1 in high-throughput, stringent Y2H screens with VAP27-1, also interacting with two of the *Hpa* effectors (HpaRXLL492 and HpaRXLL495) described in this study (Mukhtar *et al.*, 2011). VAP27-1, together with NET3C. mediates the formation of contact sites between the ER and the plasma membrane (Wang et al., 2014, 2016b) which are an attractive target for pathogen manipulation to derail intracellular communications during infection. However, our attempts to confirm the Y2H interaction between VAP27-1 and the two *Hpa* effectors by both co-immunoprecipitation and FRET-FLIM analysis were unsuccessful.

Y2H assays are context free and thus the detected effector–ANAC interactions need to be confirmed by alternative methods and, crucially, their biological relevance investigated *in planta*. This is highlighted by the observed interactions of the three mitochondria-associated effectors PITG_09216, PITG_09218, and PITG_22884 with several ER-localized ANAC TFs. It is also noted that the observed subcellular localizations of the oomycete effectors should ideally be confirmed using the native pathogen’s haustorial secretion system to directly deliver the effector into the host cell (Wang *et al.*, 2017).

In summary, our study describes a simple and rapid bioinformatic approach to identify putative ER-localized (or ER- and Golgi-localized) effectors from sequenced eukaryotic pathogens encoding a conserved C-terminal tail anchor, validating the predictions on effectors from *P. infestans*. The role of the ER in host immunity and as a target of pathogen virulence strategies has largely been ignored to date. We believe that this pipeline could be extended to other oomycete effector families, for example Crinkler (CRN), assuming that they are translocated and trafficked to their final subcellular destinations in the same way as RXLR effectors, increasing our knowledge of the extent to which the ER is targeted by oomycete effectors. The presence of a signal peptide targets the effectors for secretion by the pathogen via the conventional secretory pathway and/or via a non-conventional pathway involving extracellular vesicles (Boevink, 2017). Either way, however, it is unclear how membrane-associated proteins are subsequently trafficked to their host target, which presumably requires shielding or masking of the hydrophobic TMD during translocation. In this respect, the GET pathway provides a convenient host system for effectors to be post-translationally localized to the ER (and most probably mitochondria and chloroplast outer membranes). Therefore, a subset of pathogen effectors may have evolved tail anchors to hijack the host GET pathway, enabling specific delivery to subcellular host membranes as part of the coordinated cellular suppression of immunity.

It is now being recognized that effectors target cellular addresses other than the nucleus and cell wall, as evidenced by an increasing focus on suppression of chloroplast immunity (de Torres-Zabala *et al.*, 2015). We propose that the ER, as the major site of *de novo* lipid and protein biosynthesis, is a prime, but unappreciated, target for manipulation by pathogens orchestrated via the secretion of a suite of diverse effectors specifically targeted to this organelle.

## Supplementary data

The following supplementary data are available at *JXB* online.

Fig. S1. Unmerged confocal images of *P. halstedii* (*Ph*) and *Hpa* effectors co-expressed with the ER luminal marker (RFP–HDEL) presented in Fig. 1.

Fig. S2. Unmerged confocal images of *P. infestans* effectors co-expressed with organelle markers presented in Fig. 3.

Fig. S3. Protein–protein interactions (PPIs) between NTL TFs and *P. infestans*, *Hpa*, and *P. halstedii* effectors were determined by Y2H assays (*n*=1 of 3), as summarized in Fig. 4.

Table S1. Primers used in this study.

Table S2. Selected tail-anchored effectors from *Plasmopara halstedii* and *Hyaloperonospora arabidopsidis* (*Hpa*) characterized in this study.

Table S3. Putative tail-anchored effectors from *Phytophthora infestans* identified in this study.

Table S4. Selected tail-anchored effectors from *Bremia lactucae* characterized by Meisrimler *et al.* (2019).

erad075_suppl_Supplementary_Figures

erad075_suppl_Supplementary_Tables

## Data Availability

All data supporting the findings of this study are available within the paper and within its supplementary data published online.

## Abbreviations

BiP: Binding immunoglobulin Protein
CTS: C-terminal sequence
ER: endoplasmic reticulum
GET: guided entry of tail proteins
GFP: green fluorescent protein
GRAVY: Grand Average of Hydrophobicity
NAC: NAM (no apical meristem), ATAF1 and 2, and CUC2 (cup-shaped cotyledon)
NTL: NAC with Transmembrane Motif1-like
PCD: programmed cell death
PPI: protein–protein interaction
RFP: red fluorescent protein
TA: tail-anchored
TF: transcription factor
TMD: transmembrane domain
UPR: unfolded protein response
Y2H: yeast two-hybrid

## References

[CIT0001] Ah-Fong AMV , Kim KS, Judelson HS. 2017. RNA-seq of life stages of the oomycete *Phytophthora infestans* reveals dynamic changes in metabolic, signal transduction, and pathogenesis genes and a major role for calcium signaling in development. BMC Genomics 18, 198. 28228125 10.1186/s12864-017-3585-x PMC5322657

[CIT0002] Almagro Armenteros JJ , Salvatore M, Emanuelsson O, Winther O, von Heijne G, Elofsson A, Nielsen H. 2019a. Detecting sequence signals in targeting peptides using deep learning. Life Science Alliance 2, e201900429. 31570514 10.26508/lsa.201900429 PMC6769257

[CIT0003] Almagro Armenteros JJ , Tsirigos KD, Sønderby CK, Petersen TN, Winther O, Brunak S, Heijne G, Nielsen H. 2019b. SignalP 5.0 improves signal peptide predictions using deep neural networks. Nature Biotechnology 37, 420–423. 10.1038/s41587-019-0036-z 30778233

[CIT0004] Barton K , Mathur N, Mathur J. 2013. Simultaneous live-imaging of peroxisomes and the ER in plant cells suggests contiguity but no luminal continuity between the two organelles. Frontiers in Physiology 4, 196. 23898304 10.3389/fphys.2013.00196 PMC3721060

[CIT0005] Baxter L , Tripathy S, Ishaque N, et al. 2010. Signatures of adaptation to obligate biotrophy in the *Hyaloperonospora arabidopsidis* genome. Science 330, 1549–1551. 21148394 10.1126/science.1195203 PMC3971456

[CIT0006] Block A , Toruño TY, Elowsky CG, Zhang C, Steinbrenner J, Beynon J, Alfano JR. 2014. The *Pseudomonas syringae* type III effector HopD1 suppresses effector-triggered immunity, localizes to the endoplasmic reticulum, and targets the Arabidopsis transcription factor NTL9. New Phytologist 201, 1358–1370. 24329768 10.1111/nph.12626

[CIT0007] Boevink PC. 2017. Exchanging missives and missiles: the roles of extracellular vesicles in plant–pathogen interactions. Journal of Experimental Botany 68, 5411–5414. 29190393 10.1093/jxb/erx369 PMC5853247

[CIT0008] Boevink PC , Birch PRJ, Turnbull D, Whisson SC. 2020. Devastating intimacy: the cell biology of plant–*Phytophthora* interactions. New Phytologist 32, 1077. 10.1111/nph.16650 PMC7540312 32394464

[CIT0009] Borgese N , Colombo S, Pedrazzini E. 2003. The tale of tail-anchored proteins: coming from the cytosol and looking for a membrane. Journal of Cell Biology 161, 1013–1019. 12821639 10.1083/jcb.200303069 PMC2173004

[CIT0010] Bozkurt TO , Kamoun S. 2020. The plant–pathogen haustorial interface at a glance. Journal of Cell Science 133, jcs237958–jcs237956. 32132107 10.1242/jcs.237958 PMC7075074

[CIT0011] Brandizzi F. 2021. Maintaining the structural and functional homeostasis of the plant endoplasmic reticulum. Developmental Cell 56, 919–932. 33662257 10.1016/j.devcel.2021.02.008 PMC8922286

[CIT0012] Brandizzi F , Frangne N, Marc-Martin S, Hawes C, Neuhaus J-M, Paris N. 2002. The destination for single-pass membrane proteins is influenced markedly by the length of the hydrophobic domain. The Plant Cell 14, 1077–1092. 12034898 10.1105/tpc.000620 PMC150608

[CIT0013] Breeze E , Mullineaux PM. 2022. The passage of H_2_O_2_ from chloroplasts to their associated nucleus during retrograde signalling: reflections on the role of the nuclear envelope. Plants (Basel) 11, 552. 35214888 10.3390/plants11040552 PMC8876790

[CIT0014] Caillaud M-C , Piquerez SJM, Fabro G, Steinbrenner J, Ishaque N, Beynon J, Jones JDG. 2012. Subcellular localization of the Hpa RxLR effector repertoire identifies a tonoplast-associated protein HaRxL17 that confers enhanced plant susceptibility. The Plant Journal 69, 252–265. 21914011 10.1111/j.1365-313X.2011.04787.x

[CIT0015] Chio US , Cho H, Shan S-o. 2017. Mechanisms of tail-anchored membrane protein targeting and insertion. Annual Review of Cell and Developmental Biology 33, 417–438. 10.1146/annurev-cellbio-100616-060839 PMC6343671 28992441

[CIT0016] Costes SV , Daelemans D, Cho EH, Dobbin Z, Pavlakis G, Lockett S. 2004. Automatic and quantitative measurement of protein–protein colocalization in live cells. Biophysical Journal 86, 3993–4003. 15189895 10.1529/biophysj.103.038422 PMC1304300

[CIT0017] de Torres-Zabala M , Littlejohn G, Jayaraman S, et al. 2015. Chloroplasts play a central role in plant defence and are targeted by pathogen effectors. Nature Plants 1, 15074. 27250009 10.1038/nplants.2015.74

[CIT0018] Dong S , Yin W, Kong G, et al. 2011. *Phytophthora sojae* avirulence effector Avr3b is a secreted NADH and ADP-ribose pyrophosphorylase that modulates plant immunity. PLoS Pathogens 7, e1002353. 22102810 10.1371/journal.ppat.1002353 PMC3213090

[CIT0019] Dou D , Kale SD, Wang X, Jiang RHY, Bruce NA, Arredondo FD, Zhang X, Tyler BM. 2008. RXLR-mediated entry of *Phytophthora sojae* effector Avr1b into soybean cells does not require pathogen-encoded machinery. The Plant Cell 20, 1930–1947. 18621946 10.1105/tpc.107.056093 PMC2518231

[CIT0020] Fabro G. 2021. Oomycete intracellular effectors: specialised weapons targeting strategic plant processes. New Phytologist 233, 1074–1082. 34705271 10.1111/nph.17828

[CIT0021] Fan G , Yang Y, Li T, Lu W, Du Y, Qiang X, Wen Q, Shan W. 2018. A *Phytophthora capsici* RXLR effector targets and inhibits a plant PPIase to suppress endoplasmic reticulum-mediated immunity. Molecular Plant 11, 1067–1083. 29864524 10.1016/j.molp.2018.05.009

[CIT0022] Gomord V , Denmat LA, Fitchette-Lainé AC, Satiat-Jeunemaitre B, Hawes C, Faye L. 1997. The C-terminal HDEL sequence is sufficient for retention of secretory proteins in the endoplasmic reticulum (ER) but promotes vacuolar targeting of proteins that escape the ER. The Plant Journal 11, 313–325. 9076996 10.1046/j.1365-313x.1997.11020313.x

[CIT0023] Haas BJ , Kamoun S, Zody MC, et al. 2009. Genome sequence and analysis of the Irish potato famine pathogen *Phytophthora infestans*. Nature 461, 393–398. 19741609 10.1038/nature08358

[CIT0024] Harvey S , Kumari P, Lapin D, et al. 2020. Downy Mildew effector HaRxL21 interacts with the transcriptional repressor TOPLESS to promote pathogen susceptibility. PLoS Pathogens 16, e1008835. 32785253 10.1371/journal.ppat.1008835 PMC7446885

[CIT0025] Hawes C , Kiviniemi P, Kriechbaumer V. 2015. The endoplasmic reticulum: a dynamic and well-connected organelle. Journal of Integrative Plant Biology 57, 50–62. 25319240 10.1111/jipb.12297

[CIT0026] Hegde RS , Keenan RJ. 2011. Tail-anchored membrane protein insertion into the endoplasmic reticulum. Nature Reviews. Molecular Cell Biology 12, 787–798. 22086371 10.1038/nrm3226 PMC3760496

[CIT0027] Hossain MM , Pérez-López E, Todd CD, Wei Y, Bonham-Smith PC. 2021. Endomembrane-targeting *Plasmodiophora brassicae* effectors modulate PAMP triggered immune responses in plants. Frontiers in Microbiology 12. 10.3389/fmicb.2021.651279 PMC8282356 34276588

[CIT0028] Jiang RHY , Tripathy S, Govers F, Tyler BM. 2008. RXLR effector reservoir in two Phytophthora species is dominated by a single rapidly evolving superfamily with more than 700 members. Proceedings of the National Academy of Sciences, USA 105, 4874–4879. 10.1073/pnas.0709303105 PMC2290801 18344324

[CIT0029] Jing M , Guo B, Li H, et al. 2016. A *Phytophthora sojae* effector suppresses endoplasmic reticulum stress-mediated immunity by stabilizing plant Binding immunoglobulin Proteins. Nature Communications 7, 11685. 10.1038/ncomms11685 PMC4895818 27256489

[CIT0030] Kale SD , Gu B, Capelluto DG, et al. 2010. External lipid PI3P mediates entry of eukaryotic pathogen effectors into plant and animal host cells. Cell 142, 284–295. 20655469 10.1016/j.cell.2010.06.008

[CIT0031] Kamoun S , Furzer O, Jones JDG, et al. 2015. The top 10 oomycete pathogens in molecular plant pathology. Molecular Plant Pathology 16, 413–434. 25178392 10.1111/mpp.12190 PMC6638381

[CIT0032] Khan M , Seto D, Subramaniam R, Desveaux D. 2017. Oh, the places they’ll go! A survey of phytopathogen effectors and their host targets. The Plant Journal 93, 651–663. 29160935 10.1111/tpj.13780

[CIT0033] Kim S-G , Lee S, Seo PJ, Kim S-K, Kim J-K, Park C-M. 2010. Genome-scale screening and molecular characterization of membrane-bound transcription factors in Arabidopsis and rice. Genomics 95, 56–65. 19766710 10.1016/j.ygeno.2009.09.003

[CIT0034] Kim S-Y , Kim S-G, Kim Y-S, Seo PJ, Bae M, Yoon H-K, Park C-M. 2006. Exploring membrane-associated NAC transcription factors in Arabidopsis: implications for membrane biology in genome regulation. Nucleic Acids Research 35, 203–213. 17158162 10.1093/nar/gkl1068 PMC1802569

[CIT0035] Knapova G , Gisi U. 2002. Phenotypic and genotypic structure of *Phytophthora infestans* populations on potato and tomato in France and Switzerland. Plant Pathology 51, 641–653.

[CIT0036] Kriechbaumer V , Shaw R, Mukherjee J, Bowsher CG, Harrison A-M, Abell BM. 2009. Subcellular distribution of tail-anchored proteins in Arabidopsis. Traffic 10, 1753–1764. 19843281 10.1111/j.1600-0854.2009.00991.x

[CIT0037] Krogh A , Larsson B, von Heijne G, Sonnhammer ELL. 2001. Predicting transmembrane protein topology with a hidden Markov model: application to complete genomes. Journal of Molecular Biology 305, 567–580. 11152613 10.1006/jmbi.2000.4315

[CIT0038] Kwaaitaal M , Nielsen ME, Böhlenius H, Thordal-Christensen H. 2017. The plant membrane surrounding powdery mildew haustoria shares properties with the endoplasmic reticulum membrane. Journal of Experimental Botany 68, 5731–5743. 29237056 10.1093/jxb/erx403 PMC5854130

[CIT0039] Kyte J , Doolittle RF. 1982. A simple method for displaying the hydropathic character of a protein. Journal of Molecular Biology 157, 105–132. 7108955 10.1016/0022-2836(82)90515-0

[CIT0040] Letunic I , Bork P. 2019. Interactive Tree Of Life (iTOL) v4: recent updates and new developments. Nucleic Acids Research 47, W256–W259. 30931475 10.1093/nar/gkz239 PMC6602468

[CIT0041] Lewis LA , Polanski K, de Torres-Zabala M, et al. 2015. Transcriptional dynamics driving MAMP-triggered immunity and pathogen effector-mediated immunosuppression in Arabidopsis leaves following infection with *Pseudomonas syringae* pv tomato DC3000. The Plant Cell 27, 3038–3064. 26566919 10.1105/tpc.15.00471 PMC4682296

[CIT0042] Liang M , Li H, Zhou F, Li H, Liu J, Hao Y, Wang Y, Zhao H, Han S. 2015. Subcellular distribution of NTL transcription factors in *Arabidopsis thaliana*. Traffic 16, 1062–1074. 26201836 10.1111/tra.12311

[CIT0043] Liu J , Chen S, Ma T, Gao Y, Song S, Ye W, Lu J. 2021. *Plasmopara viticola* effector PvRXLR53 suppresses innate immunity in *Nicotiana benthamiana*. Plant Signaling & Behavior 16, 1846927. 33210976 10.1080/15592324.2020.1846927 PMC7849728

[CIT0044] Liu JX , Howell SH. 2010. Endoplasmic reticulum protein quality control and its relationship to environmental stress responses in plants. The Plant Cell 22, 2930–2942. 20876830 10.1105/tpc.110.078154 PMC2965551

[CIT0045] Liu JX , Howell SH. 2016. Managing the protein folding demands in the endoplasmic reticulum of plants. New Phytologist 211, 418–428. 26990454 10.1111/nph.13915

[CIT0046] Liu Y , Lan X, Song S, Yin L, Dry IB, Qu J, Xiang J, Lu J. 2018. In planta functional analysis and subcellular localization of the oomycete pathogen *Plasmopara viticola* candidate RXLR effector repertoire. Frontiers in Plant Science 9, 286. 29706971 10.3389/fpls.2018.00286 PMC5908963

[CIT0047] Marty NJ , Teresinski HJ, Hwang YT, et al. 2014. New insights into the targeting of a subset of tail-anchored proteins to the outer mitochondrial membrane. Frontiers in Plant Science 5, 426. 25237314 10.3389/fpls.2014.00426 PMC4154396

[CIT0048] McCarthy CGP , Fitzpatrick DA. 2017. Phylogenomic reconstruction of the oomycete phylogeny derived from 37 genomes. mSphere 2, 3–17. 10.1128/mSphere.00095-17 PMC5390094 28435885

[CIT0049] McLellan H , Boevink PC, Armstrong MR, Pritchard L, Gomez S, Morales J, Whisson SC, Beynon JL, Birch PRJ. 2013. An RxLR effector from *Phytophthora infestan*s prevents re-localisation of two plant NAC transcription factors from the endoplasmic reticulum to the nucleus. PLoS Pathogens 9, e1003670. 24130484 10.1371/journal.ppat.1003670 PMC3795001

[CIT0050] McLellan H , Harvey SE, Steinbrenner J, et al. 2022. Exploiting breakdown in nonhost effector–target interactions to boost host disease resistance. Proceedings of the National Academy of Sciences, USA 119, e2114064119. 10.1073/pnas.2114064119 PMC9436328 35994659

[CIT0051] Mehrshahi P , Stefano G, Andaloro JM, Brandizzi F, Froehlich JE, DellaPenna D. 2013. Transorganellar complementation redefines the biochemical continuity of endoplasmic reticulum and chloroplasts. Proceedings of the National Academy of Sciences, USA 110, 12126–12131. 10.1073/pnas.1306331110 PMC3718160 23818635

[CIT0052] Meisrimler CN , Pelgrom AJE, Oud B, Out S, Van den Ackerveken G. 2019. Multiple downy mildew effectors target the stress‐related NAC transcription factor LsAC069 in lettuce. The Plant Journal 99, 1098–1115. 31077456 10.1111/tpj.14383 PMC9545932

[CIT0053] Morishita T , Kojima Y, Maruta T, Nishizawa-Yokoi A, Yabuta Y, Shigeoka S. 2009. Arabidopsis NAC transcription factor, ANAC078, regulates flavonoid biosynthesis under high-light. Plant and Cell Physiology 50, 2210–2222. 19887540 10.1093/pcp/pcp159

[CIT0054] Mukhtar MS , Carvunis A-R, Dreze M, et al. 2011. Independently evolved virulence effectors converge onto hubs in a plant immune system network. Science 333, 596–601. 21798943 10.1126/science.1203659 PMC3170753

[CIT0055] Nakamura S , Mano S, Tanaka Y, et al. 2014. Gateway binary vectors with the bialaphos resistance gene, bar, as a selection marker for plant transformation. Bioscience, Biotechnology, and Biochemistry 74, 1315–1319. 10.1271/bbb.100184 20530878

[CIT0056] Nelson BK , Cai X, Nebenführ A. 2007. A multicolored set of in vivo organelle markers for co-localization studies in Arabidopsis and other plants. The Plant Journal 51, 1126–1136. 17666025 10.1111/j.1365-313X.2007.03212.x

[CIT0057] O’Connell RJ , Panstruga R. 2006. Tete a tete inside a plant cell: establishing compatibility between plants and biotrophic fungi and oomycetes. New Phytologist 171, 699–718. 16918543 10.1111/j.1469-8137.2006.01829.x

[CIT0058] Oliveira-Garcia E , Tamang TM, Park J, Dalby M, Martin-Urdiroz M, Herrero CR, Vu AH, Park S, Talbot NJ, Valent B. 2022. Clathrin-mediated endocytosis facilitates internalization of magnaporthe oryzae effectors into rice cells. bioRxiv. doi:10.1101/2021.12.28.474284. [Preprint]. PMC10291035 36976907

[CIT0059] Parsons HT , Stevens TJ, McFarlane HE, et al. 2019. Separating Golgi proteins from cis to trans reveals underlying properties of cisternal localization. The Plant Cell 31, 2010–2034. 31266899 10.1105/tpc.19.00081 PMC6751122

[CIT0060] Pastor-Cantizano N , Ko DK, Angelos E, Pu Y, Brandizzi F. 2020. Functional diversification of ER stress responses in arabidopsis. Trends in Biochemical Sciences 45, 123–136. 31753702 10.1016/j.tibs.2019.10.008 PMC6980780

[CIT0061] Pecrix Y , Buendia L, Penouilh Suzette C, et al. 2019. Sunflower resistance to multiple downy mildew pathotypes revealed by recognition of conserved effectors of the oomycete *Plasmopara halstedii*. The Plant Journal 97, 730–748. 30422341 10.1111/tpj.14157 PMC6849628

[CIT0062] Pérez-Sancho J , Tilsner J, Samuels AL, Botella MA, Bayer EM, Rosado A. 2016. Stitching organelles: organization and function of specialized membrane contact sites in plants. Trends in Cell Biology 26, 705–717. 27318776 10.1016/j.tcb.2016.05.007

[CIT0063] Perico C , Sparkes I. 2018. Plant organelle dynamics: cytoskeletal control and membrane contact sites. New Phytologist 282, 1170–1114. 10.1111/nph.15365 30078196

[CIT0064] Petre B , Contreras MP, Bozkurt TO, et al. 2021. Host–interactor screens of *Phytophthora infestans* RXLR proteins reveal vesicle trafficking as a major effector-targeted process. The Plant Cell 33, 1447–1471. 33677602 10.1093/plcell/koab069 PMC8254500

[CIT0065] Petre B , Kamoun S. 2014. How do filamentous pathogens deliver effector proteins into plant cells? PLoS Biology 12, e1001801. 24586116 10.1371/journal.pbio.1001801 PMC3934835

[CIT0066] Rao M , Okreglak V, Chio US, Cho H, Walter P, Shan S-o. 2016. Multiple selection filters ensure accurate tail-anchored membrane protein targeting. eLife 5, 1743. 10.7554/eLife.21301 PMC5214336 27925580

[CIT0067] Savage Z , Duggan C, Toufexi A, et al. 2021. Chloroplasts alter their morphology and accumulate at the pathogen interface during infection by *Phytophthora infestans*. The Plant Journal 107, 1771–1787. 34250673 10.1111/tpj.15416

[CIT0068] Schutze MP , Peterson PA, Jackson MR. 1994. An N-terminal double-arginine motif maintains type II membrane proteins in the endoplasmic reticulum. The EMBO Journal 13, 1696–1705. 8157008 10.1002/j.1460-2075.1994.tb06434.x PMC395002

[CIT0069] Sharma R , Xia X, Cano LM, et al. 2015. Genome analyses of the sunflower pathogen *Plasmopara halstedii* provide insights into effector evolution in downy mildews and *Phytophthora*. BMC Genomics 16, 741. 26438312 10.1186/s12864-015-1904-7 PMC4594904

[CIT0070] Sievers F , Wilm A, Dineen D, et al. 2011. Fast, scalable generation of high-quality protein multiple sequence alignments using Clustal Omega. Molecular Systems Biology 7, 1–6. 10.1038/msb.2011.75 PMC3261699 21988835

[CIT0071] Sparkes IA , Ketelaar T, de Ruijter NCA, Hawes C. 2009. Grab a Golgi: laser trapping of Golgi bodies reveals in vivo interactions with the endoplasmic reticulum. Traffic 10, 567–571. 19220813 10.1111/j.1600-0854.2009.00891.x

[CIT0072] Sparkes IA , Runions J, Kearns A, Hawes C. 2006. Rapid, transient expression of fluorescent fusion proteins in tobacco plants and generation of stably transformed plants. Nature Protocols 1, 2019–2025. 17487191 10.1038/nprot.2006.286

[CIT0073] Sperschneider J , Williams AH, Hane JK, Singh KB, Taylor JM. 2015. Evaluation of secretion prediction highlights differing approaches needed for oomycete and fungal effectors. Frontiers in Plant Science 6, 1168. 26779196 10.3389/fpls.2015.01168 PMC4688413

[CIT0074] Srivastava R , Li Z, Russo G, et al. 2018. Response to persistent ER stress in plants: a multiphasic process that transitions cells from prosurvival activities to cell death. The Plant Cell 30, 1220–1242. 29802214 10.1105/tpc.18.00153 PMC6048783

[CIT0075] Srivastava R , Zalisko BE, Keenan RJ, Howell SH. 2017. The GET system inserts the tail-anchored protein, SYP72, into endoplasmic reticulum membranes. Plant Physiology 173, 1137–1145. 27923985 10.1104/pp.16.00928 PMC5291014

[CIT0076] Stefano G , Renna L, Lai Y, Slabaugh E, Mannino N, Buono RA, Otegui MS, Brandizzi F. 2015. ER network homeostasis is critical for plant endosome streaming and endocytosis. Cell Discovery 1, 15033. 27462431 10.1038/celldisc.2015.33 PMC4860783

[CIT0077] Takemoto D , Jones DA, Hardham AR. 2003. GFP-tagging of cell components reveals the dynamics of subcellular re-organization in response to infection of Arabidopsis by oomycete pathogens. The Plant Journal 33, 775–792. 12609049 10.1046/j.1365-313x.2003.01673.x

[CIT0078] Teresinski HJ , Gidda SK, Nguyen TND, Howard NJM, Porter BK, Grimberg N, Smith MD, Andrews DW, Dyer JM, Mullen RT. 2018. An RK/ST C-terminal motif is required for targeting of OEP7.2 and a subset of other arabidopsis tail-anchored proteins to the plastid outer envelope membrane. Plant and Cell Physiology 60, 516–537. 10.1093/pcp/pcy234 30521026

[CIT0079] Tsirigos KD , Peters C, Shu N, Käll L, Elofsson A. 2015. The TOPCONS web server for consensus prediction of membrane protein topology and signal peptides. Nucleic Acids Research 43, W401–W407. 25969446 10.1093/nar/gkv485 PMC4489233

[CIT0080] Tyler BM , Tripathy S, Zhang X, et al. 2006. Phytophthora genome sequences uncover evolutionary origins and mechanisms of pathogenesis. Science 313, 1261–1266. 16946064 10.1126/science.1128796

[CIT0081] Voorhees RM , Hegde RS. 2016. Toward a structural understanding of co-translational protein translocation. Current Opinion in Cell Biology 41, 91–99. 27155805 10.1016/j.ceb.2016.04.009

[CIT0082] Wang P , Hawes C, Hussey PJ. 2016a. Plant endoplasmic reticulum–plasma membrane contact sites. Trends in Plant Science, 1–10. 10.1016/j.tplants.2016.11.008 27955928

[CIT0083] Wang P , Hawkins TJ, Richardson C, Cummins I, Deeks MJ, Sparkes I, Hawes C, Hussey PJ. 2014. The plant cytoskeleton, NET3C, and VAP27 mediate the link between the plasma membrane and endoplasmic reticulum. Current Biology 24, 1397–1405. 24909329 10.1016/j.cub.2014.05.003

[CIT0084] Wang P , Richardson C, Hawkins TJ, Sparkes I, Hawes C, Hussey PJ. 2016b. Plant VAP27 proteins: domain characterization, intracellular localization and role in plant development. New Phytologist 210, 1311–1326. 27159525 10.1111/nph.13857

[CIT0085] Wang S , Boevink PC, Welsh L, Zhang R, Whisson SC, Birch PRJ. 2017. Delivery of cytoplasmic and apoplastic effectors from *Phytophthora infestans* haustoria by distinct secretion pathways. New Phytologist 216, 205–215. 28758684 10.1111/nph.14696 PMC5601276

[CIT0086] Wang S , McLellan H, Bukharova T, et al. 2019. *Phytophthora infestans* RXLR effectors act in concert at diverse subcellular locations to enhance host colonization. Journal of Experimental Botany 70, 343–356. 30329083 10.1093/jxb/ery360 PMC6305197

[CIT0087] Wang Y , Tyler BM, Wang Y. 2019b. Defense and counterdefense during plant-pathogenic oomycete infection. Annual Review of Microbiology 73, 667–696. 10.1146/annurev-micro-020518-120022 31226025

[CIT0088] Weßling R , Epple P, Altmann S, et al. 2014. Convergent targeting of a common host protein-network by pathogen effectors from three kingdoms of life. Cell Host and Microbe 16, 364–375. 25211078 10.1016/j.chom.2014.08.004 PMC4191710

[CIT0089] Whisson SC , Boevink PC, Moleleki L, et al. 2007. A translocation signal for delivery of oomycete effector proteins into host plant cells. Nature 450, 115–118. 17914356 10.1038/nature06203

[CIT0090] White RR , Lin C, Leaves I, Castro IG, Metz J, Bateman BC, Botchway SW, Ward AD, Ashwin P, Sparkes I. 2020. Miro2 tethers the ER to mitochondria to promote mitochondrial fusion in tobacco leaf epidermal cells. Communications Biology 3, 161. 32246085 10.1038/s42003-020-0872-x PMC7125145

[CIT0091] Windram O , Madhou P, McHattie S, et al. 2012. Arabidopsis defense against *Botrytis cinerea*: chronology and regulation deciphered by high-resolution temporal transcriptomic analysis. The Plant Cell 24, 3530–3557. 23023172 10.1105/tpc.112.102046 PMC3480286

[CIT0092] Xing S , Mehlhorn DG, Wallmeroth N, et al. 2017. Loss of GET pathway orthologs in *Arabidopsis thaliana* causes root hair growth defects and affects SNARE abundance. Proceedings of the National Academy of Sciences, USA 114, E1544–E1553. 10.1073/pnas.1619525114 PMC5338382 28096354

[CIT0093] Yang Z-T , Wang M-J, Sun L, Lu S-J, Bi D-L, Sun L, Song Z-T, Zhang S-S, Zhou S-F, Liu J-X. 2014. The membrane-associated transcription factor NAC089 controls ER-stress-induced programmed cell death in plants. PLoS Genetics 10, e1004243. 24675811 10.1371/journal.pgen.1004243 PMC3967986

[CIT0094] Yin J , Gu B, Huang G, Tian Y, Quan J, Lindqvist-Kreuze H, Shan W. 2017. Conserved RXLR effector genes of *Phytophthora infestans* expressed at the early stage of potato infection are suppressive to host defense. Frontiers in Plant Science 8, 1957–1911. 29312401 10.3389/fpls.2017.02155 PMC5742156

[CIT0095] Zuluaga AP , Vega-Arreguín JC, Fei Z, Ponnala L, Lee SJ, Matas AJ, Patev S, Fry WE, Rose JKC. 2016. Transcriptional dynamics of *Phytophthora infestans* during sequential stages of hemibiotrophic infection of tomato. Molecular Plant Pathology 17, 29–41. 25845484 10.1111/mpp.12263 PMC6638332

