# Supplementary material for: A tell tail sign: a conserved C-terminal tail-anchor domain targets a subset of pathogen effectors to the plant endoplasmic reticulum
Source: J Exp Bot. 2023 Mar 1;74(10):3188–202. doi: 10.1093/jxb/erad075 (PMC10199128; doi:10.1093/jxb/erad075)
Supplement: erad075_suppl_Supplementary_Figures [file erad075_suppl_supplementary_figures.pdf]

Supplementary Figure S1

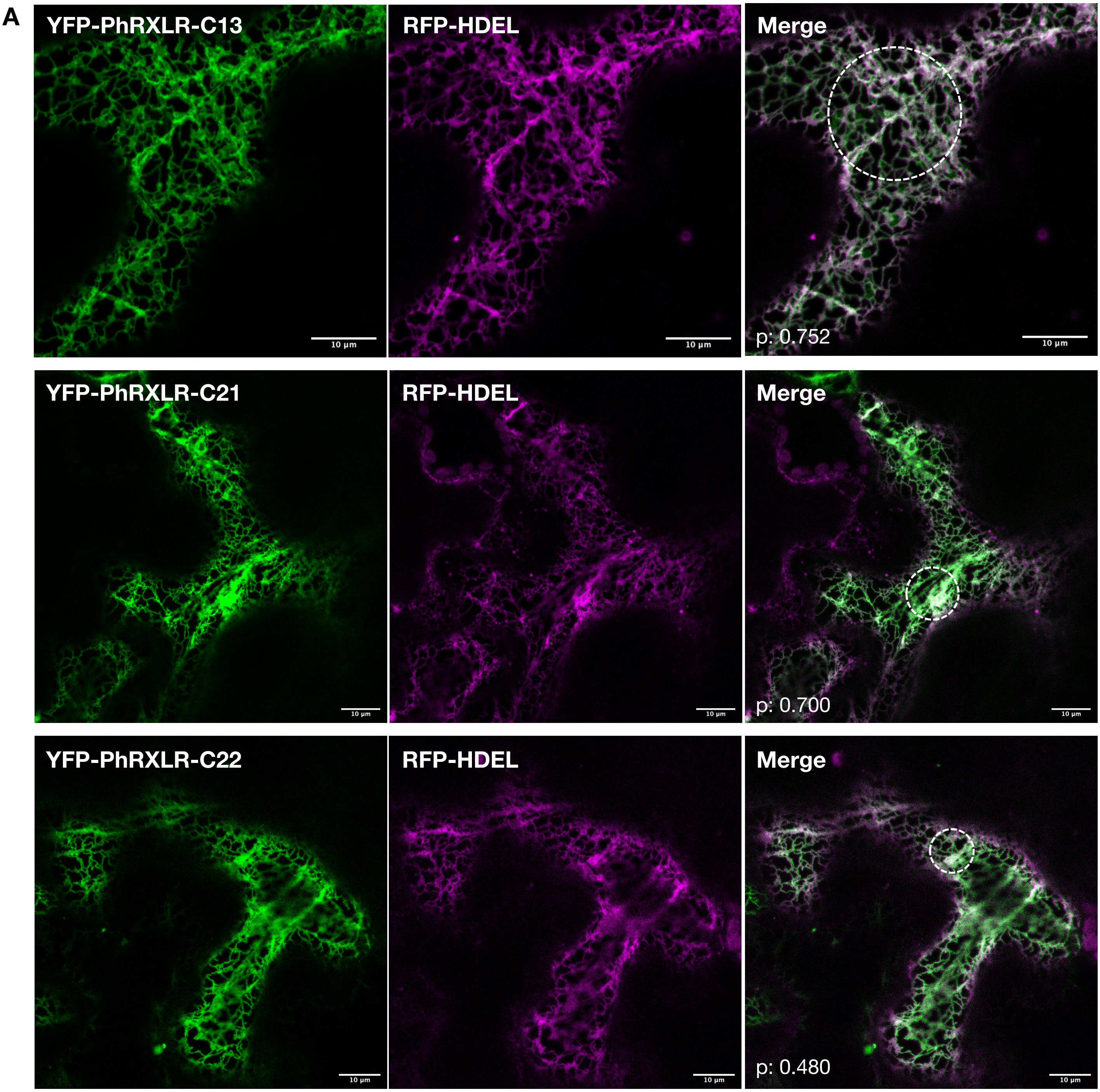

Supplementary Figure S1

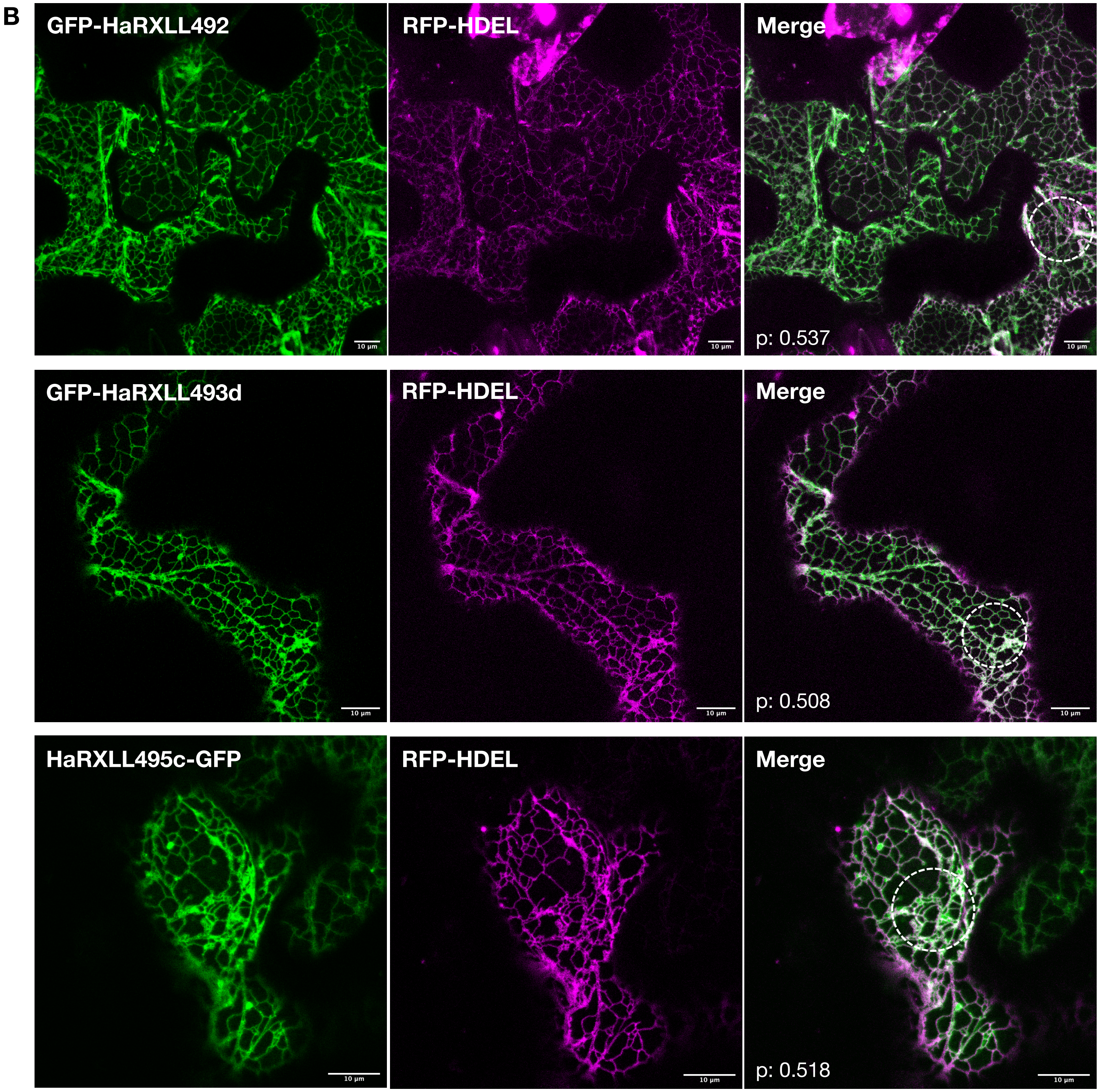

Supplementary Figure S1

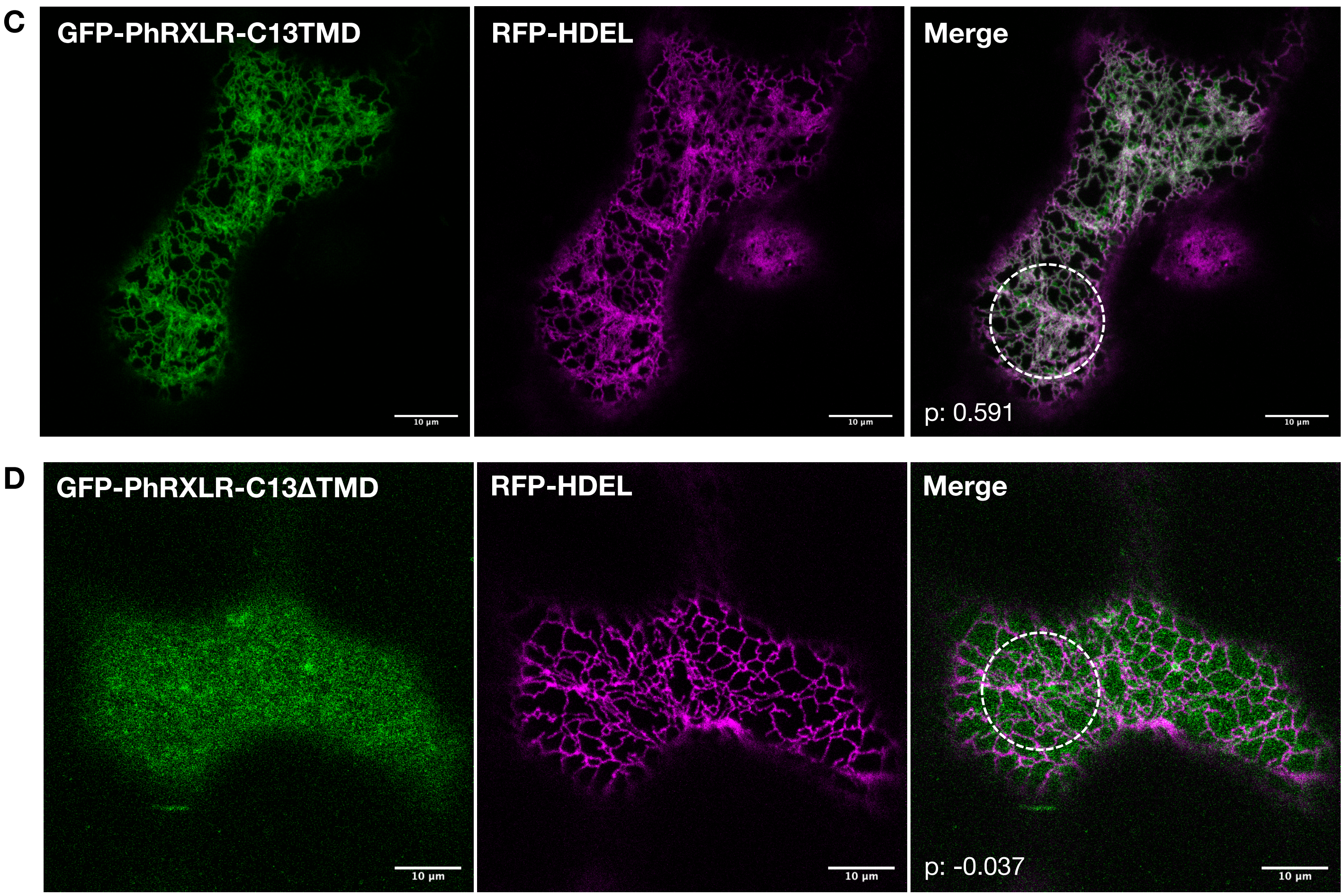

Supplementary Figure S1. Unmerged confocal images of *P. halstedii* (*Ph*) and *Hpa* effectors co-expressed with ER luminal marker (RFP-HDEL) presented in Figure 1. (A) PhRXLR-C13, C21 and C22. (B) HpaRXLL492, 493d and 495a. (C) PhRXLR-C13TMD$_{108\text{-}125.}$ (D) PhRXLR-C13ΔTMD$_{108\text{-}127}$. Colocalization scores (p: Pearson's Colocalization Coefficient) for region of interest (dashed-line circle) are indicated in overlay image (n= 3-7 cells).

Supplementary Figure S2

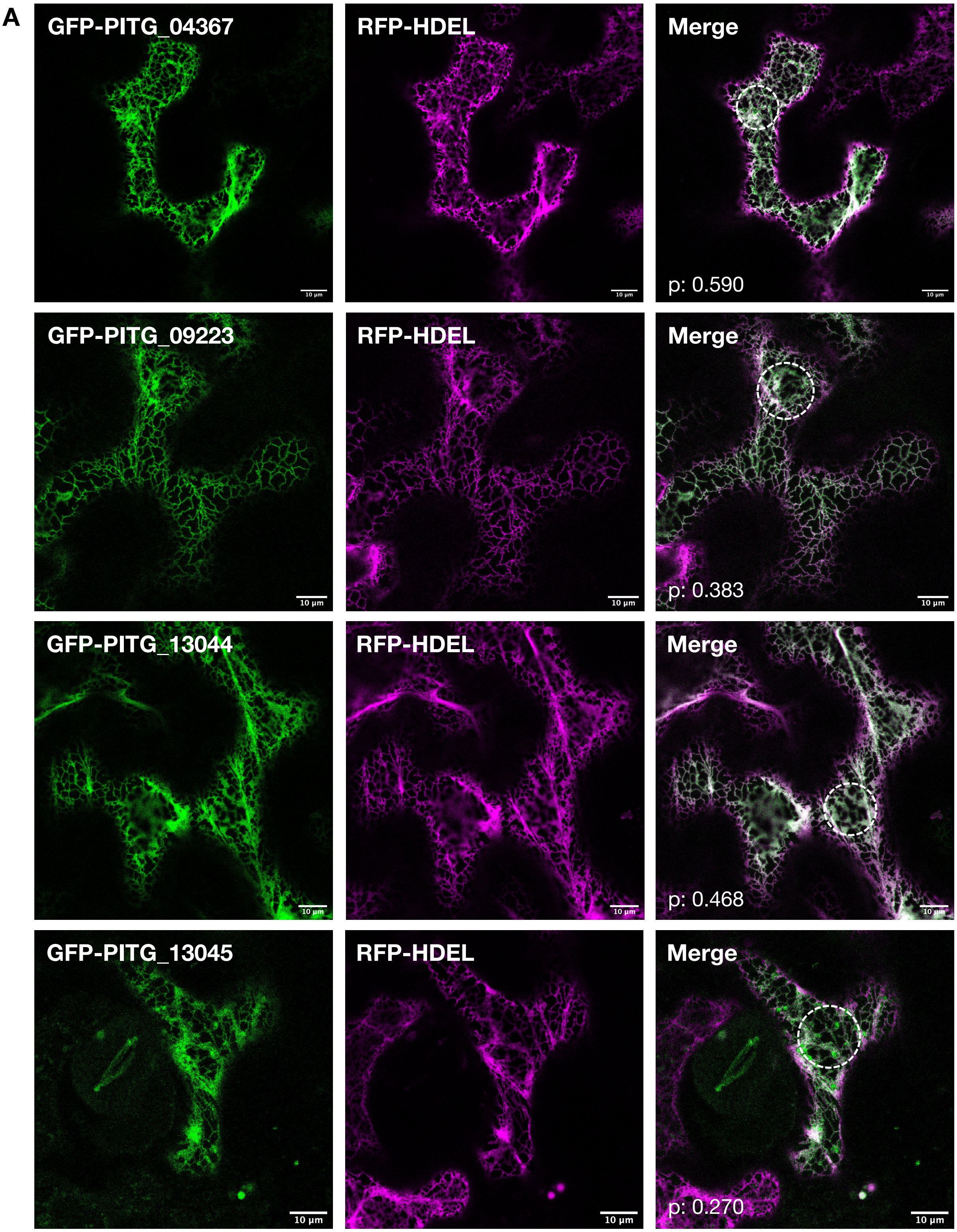

Supplementary Figure S2
A cont.

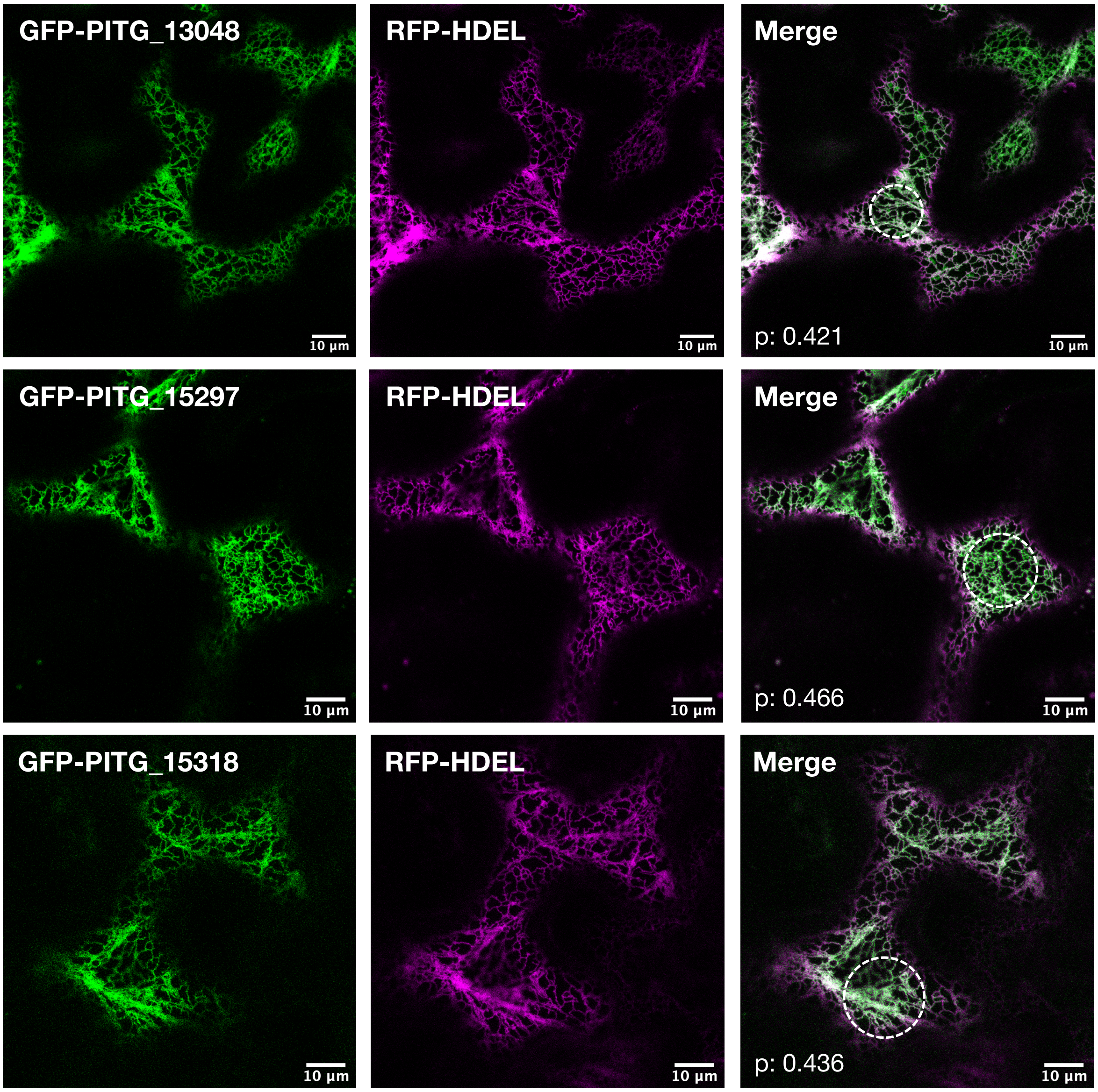

Supplementary Figure S2

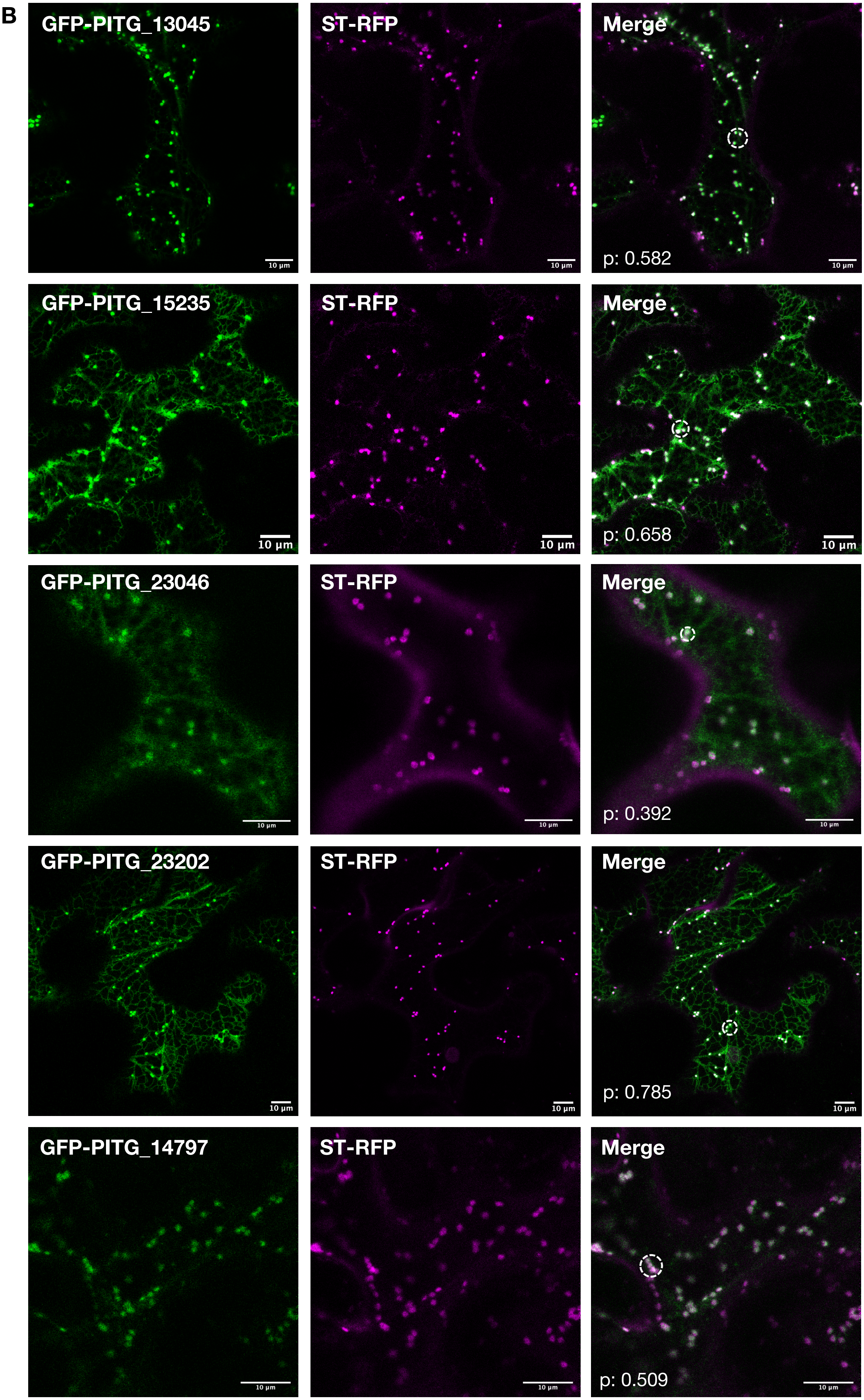

Supplementary Figure S2

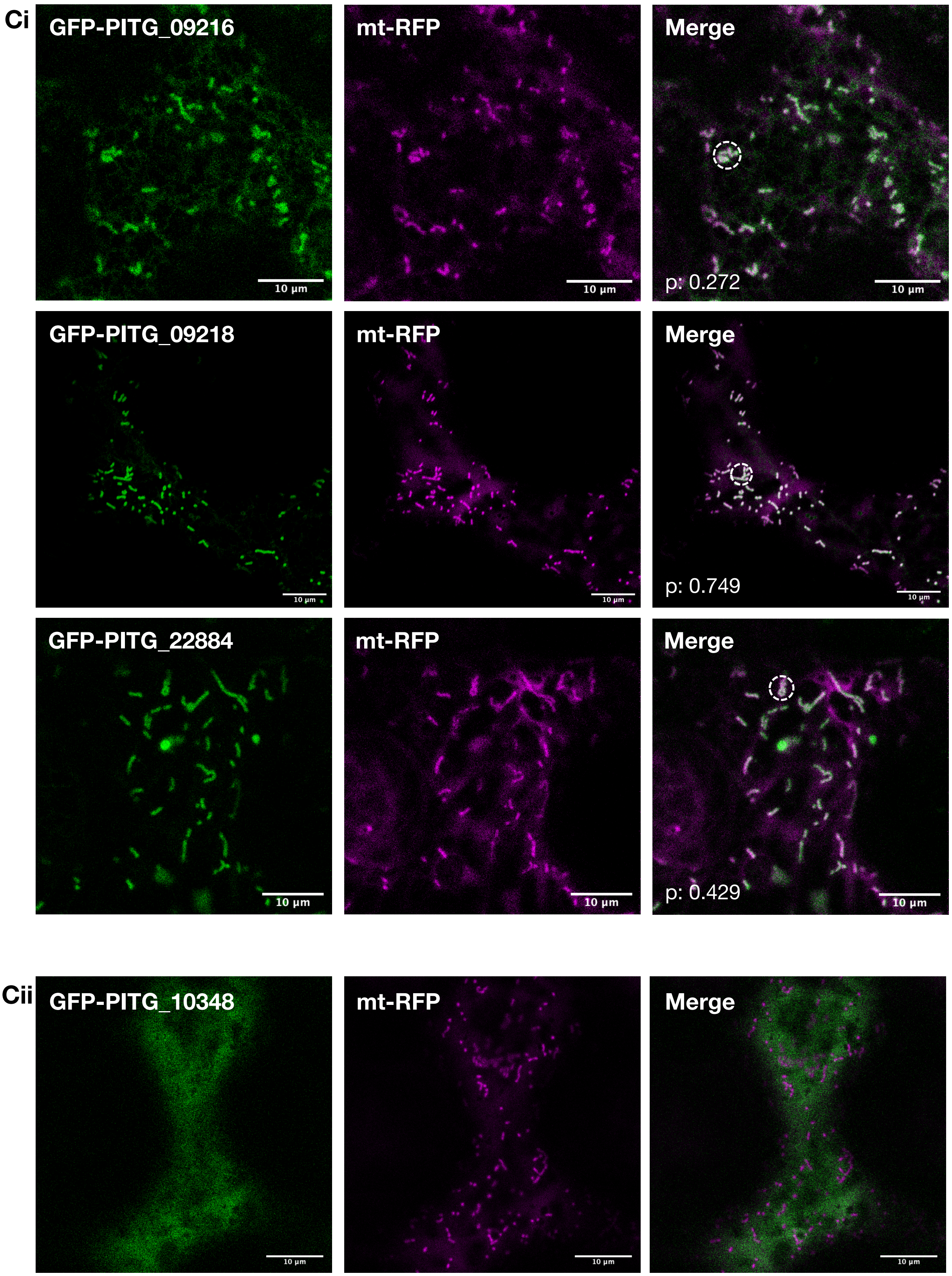

Supplementary Figure S2. Unmerged confocal images of *P. infestans* effectors co-expressed with organelle markers presented in Figure 3. (A) ER (RFP-HDEL), (B) Golgi (ST-RFP) or (C) mitochondrial markers (mt-rk). Colocalization scores (p: Pearson's Colocalization Coefficient) for region of interest (dashed-line circle) are indicated in overlay image (n= 2-6 cells). (Cii) GFP-PITG_10348 does not colocalise with the mitochondrial marker.

Supplementary Figure S3

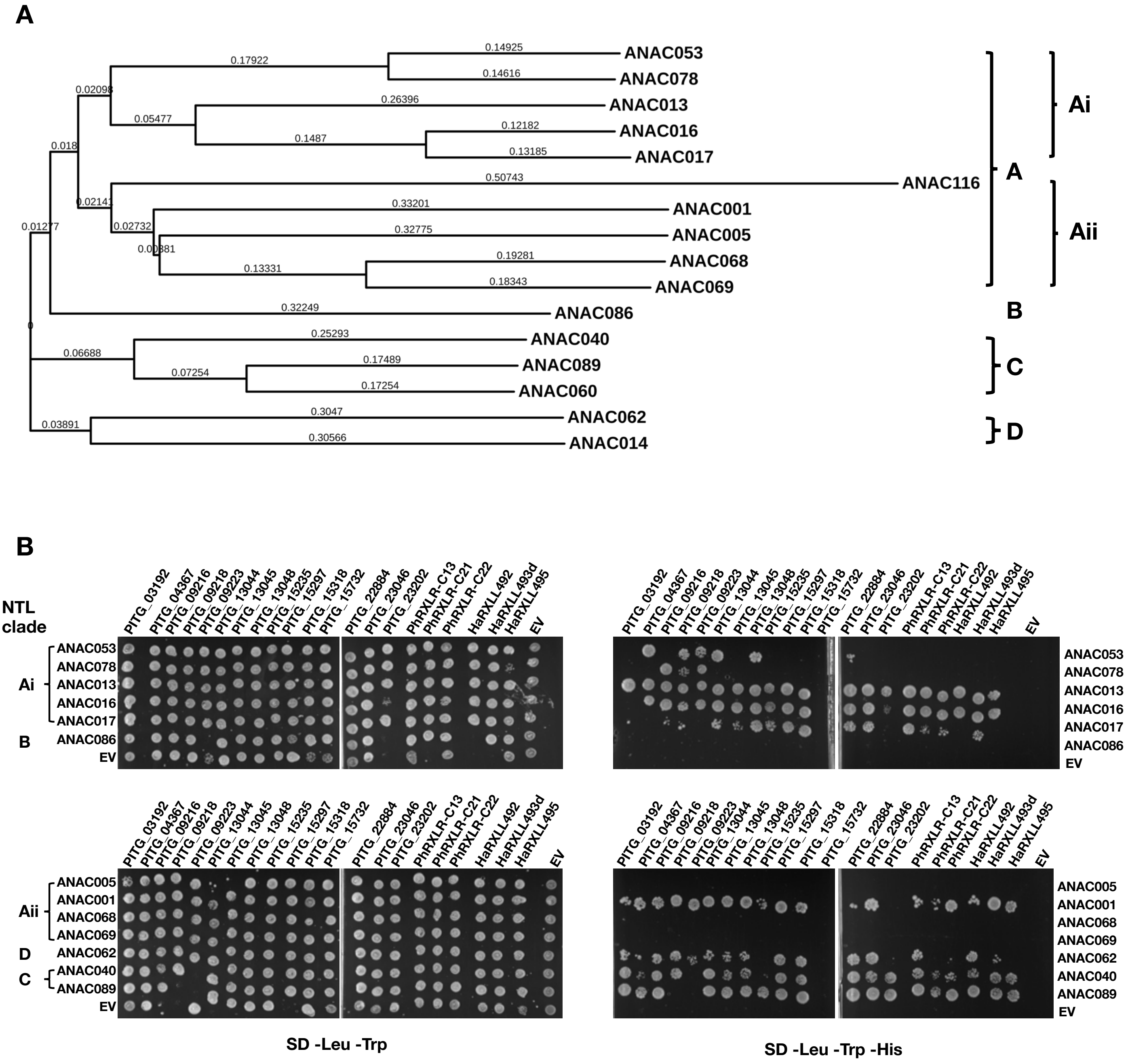

Supplementary Figure S3. Protein-protein interactions (PPIs) between NTL TFs and *P. infestans*, *Hpa* and *P. halstedii* effectors were determined by Y2H assays. (A) Phylogenetic tree of Arabidopsis NTL (ANAC) TFs, split into clades A-D. (B) Yeast growth on selective media in Y2H assay (n= 1 of 3). Positive interaction between bait constructs (effector-GAL4 binding domain fusion) and prey constructs (NAC-GAL4 activation domain fusion) resulting in activation of the HIS3 reporter gene were detected by growth on media lacking histidine (SD-Leu-Trp-His). Growth on SD-Leu-Trp media indicates the presence of both constructs. EV, empty pDEST22 (GAL4 activation domain) or pDEST32 (GAL4 binding domain) vector. Y2H data from all biological replicates is summarised in Figure 4 (n= 3).
